# Early female germline development in *Xenopus laevis*: Stem cells, nurse cells, and germline cysts

**DOI:** 10.1073/pnas.2522343122

**Published:** 2025-11-10

**Authors:** Asya Davidian, Allan C. Spradling

**Affiliations:** ^a^HHMI, Baltimore, MD 21218; ^b^Biosphere Sciences Division, Carnegie Institution for Science, Baltimore, MD 21218

**Keywords:** *Xenopus*, ovary, germ cell, germline stem cell, germline cyst

## Abstract

In Drosophila, germline cysts arise through synchronous mitotic divisions and acquire a polarized architecture organized by the fusome, which guides oocyte specification and supports meiotic progression. Similar cyst structures exist in nonmammalian vertebrate ovaries, but their polarity and function have remained uncertain. Using single-cell RNA sequencing and high-resolution imaging, we reconstructed the germ cell differentiation trajectory in *Xenopus laevis* and uncovered striking parallels with invertebrate and mouse cyst development. We identified a distinct germline stem cell (GSC) population marked by *piwil4*, low translational activity, and expression of neuronal-specific and transposon-silencing genes. Downstream from GSCs, during cyst development, an asymmetric fusome-like structure composed of stable microtubules forms a rosette-like connection between cystocytes and contains Golgi vesicles and endoplasmic reticulum, suggesting polarized trafficking. In contrast to previous claims, ~80% of cyst cells turned over rather than forming oocytes, consistent with a nurse cell fate. The striking parallels described here between cyst and fusome formation, polarization, cyst breakdown, and nurse-like cell turnover to produce relatively few oocytes argue that amphibian cysts have important functions in female gametogenesis.

## Significance

Here we describe the structure and development of *Xenopus* female germline cells and show their development begins with a germline stem cell (GSC). These distinctive cells selectively express genes like *piwil4* and neuronal genes such as *chgb*, suggesting a role of neuropeptides in GSC function. Unlike mouse germline cysts that generate mostly nurse cells and 4 to 6 oocytes, lower vertebrate cysts are reported to develop few or no nurse cells. We find that *Xenopus* cysts contain a microtubule-rich structure similar to the Drosophila fusome and that at least 80% of cyst cells develop as nurse cells that turn over prior to follicle formation, supporting a conserved role for polarized cysts in vertebrate oogenesis.

A stereotyped program of synchronous mitotic cell cycles with incomplete cytokinesis that produces germline cysts arose at the dawn of animal evolution and remains a feature of male gametes in all animal groups. Female gametogenesis also utilizes germline cysts in humans, other mammals, as well as many other vertebrate and invertebrate groups (1–5). In Drosophila, cysts arise from germline stem cells (GSCs) located at the anterior tip of the germarium. GSCs divide asymmetrically to produce a cystoblast, which then undergoes four synchronized divisions to generate a 16-cell cyst linked by a cytoskeleton-rich structure known as the fusome, that passes through the ring canals joining the cells. Cysts remain during early meiotic prophase in females, where they give rise, in addition to oocytes, to a second cell type, nurse cells, that are known or proposed to contribute to oocyte development. In Drosophila and other higher insects, cysts have been recognized for more than 100 y, and genetic studies show Drosophila cysts are essential for oocyte production and fertility.

The biology of the Drosophila fusome has been extensively studied (6–8) and compared to other insects (9). The fusome arises from mitotic spindle remnants during cyst formation and segregates asymmetrically at each division reflecting the developing polarity of the cyst. At the beginning of meiosis, the fusome reaches its maximum diameter and changes morphologically and in protein content throughout meiotic prophase (10, 11). The fusome turns over at the time of follicle formation, after ushering the movement of organelles and other materials from the nurse cells into the oocyte and its forming Balbiani body (Bb) (12, 13).

Three types of cyst functions have been identified. Nurse cells acquired their name from higher insects, where they synthesize and transfer nearly all oocyte materials. Male and female germline cysts in a wide range of species also serve a defensive function by sharing small RNAs targeting transposable elements and other nuclear parasites (14–16). Finally, germ cell rejuvenation processes needed for species perpetuation take place during meiosis in *Saccharomyces cerevisiae* (17) and probably all eukaryotes. Metazoans likely carry out major aspects of rejuvenation during meiosis in female gametogenesis. Drosophila germline cysts facilitate the rejuvenation of mitochondria, and potentially other organelles that are collected in the Bb (8, 12–13, 18–22).

Studies of *Xenopus* have contributed immensely to our understanding of oogenesis and its role in embryonic development. *Xenopus* oogenesis has been used to study rDNA amplification and many other aspects of oocyte development and lampbrush chromosome (LBC) function (23–29). Germ cell development in *Xenopus*, compared to other well-studied organisms, is slower; therefore, it provides a unique opportunity to study the different stages of development in detail. Also, *Xenopus* oocytes form a prominent Bb that initially arises at stage 1 and like the zebrafish Bb persists for much of oogenesis, unlike the transient Bbs of mice and Drosophila. The frog Bb plays a role in localizing messenger RNAs (mRNAs) initially at the vegetal pole that contribute to embryo patterning and to germ plasm formation (30–31). Studies of germline cysts in *Xenopus* ovaries were pioneered by Kloc et al. (32) who described 16-cell cysts, formed in synchronous mitotic divisions, containing typical ring canals as visualized by electron microscopy. Serial section reconstruction of developing cysts showed possible evidence of a fusome-like structure (FLS). However, *Xenopus* cysts were not observed to undergo apoptosis leading the authors to speculate that all cyst cells develop into oocytes.

The existence of GSCs in the *Xenopus* ovary remains unresolved. Although a unique cell type with a large nucleus and mitochondrial cloud, termed the “primary oogonial cell,” was described decades ago (25, 33), the prevailing view is that amphibians, like mammals, produce a finite pool of oocytes early in life (34–36). Small oogonial patches in adult Rana ovaries have been reported but are considered nonfunctional (35). In contrast, adult GSCs sustaining oogenesis have been well documented in zebrafish and medaka (37–39), and recent single-cell RNA sequencing (scRNA-seq) studies have enabled their molecular identification with markers such as *nanos2*, *dnmt3bb.1*, *chgb,* and *id4* (39).

Germline cysts have been identified in mice (40), *Xenopus* (32), fish (18), and birds (41, 42), and in mice, they produce both oocytes and nurse cells (8, 43). Nonetheless, the idea that nonmammalian vertebrates generate polarized germline cysts to specify oocytes and nurse cells, and to support oocyte development has remained controversial (8). Rather, the idea that vertebrate cysts are present but have no conserved function continues to be maintained, especially in the case of lower vertebrates such as amphibians and fish (5).

We examined female germ cell development in *Xenopus laevis* to look for GSCs and to better characterize amphibian germline cysts. Using single-cell transcriptomics and high-resolution microscopy, we identified GSCs marked by expression of *piwil4*, as well as neuronal gene signatures. Germ cells form cysts that assemble a FLS suggesting the emergence of early structural asymmetry. Furthermore, the vast majority of cyst cells do not become oocytes, but resemble nurse cells, persisting transiently and displaying low UMI (unique molecular identifiers) similar to mouse nurse cells (44). However, organelle bulk transfer was not observed. These findings support a largely conserved role for germline cysts and nurse cells in *Xenopus* and suggest that early cyst polarity, possible selective oocyte formation, and nurse-like cell turnover are ancestral features of female gametogenesis across animals.

## Results

### Germline Differentiation Trajectory Revealed by scRNA-seq and Cytological Markers.

**Fig. 1.**
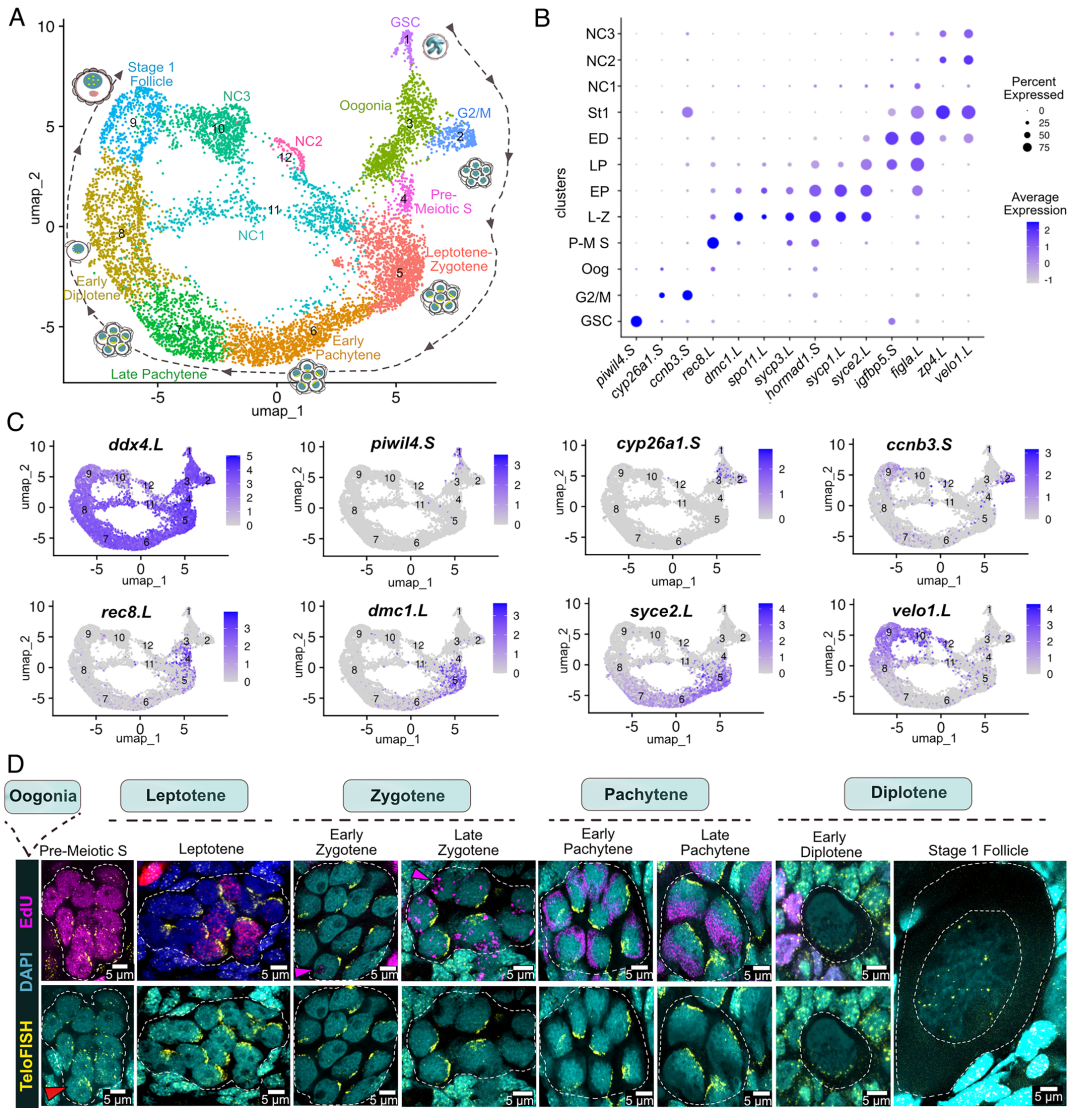
Development of cysts based on scRNAseq and telomere FISH. (*A*) UMAP visualization of germline scRNA-seq clusters from three juvenile *Xenopus* ovary replicates. Development progresses clockwise along an outer ring, from GSCs, to proliferating oogonia, premeiotic S-phase, leptotene, zygotene, pachytene, early diplotene primordial follicles, and stage I follicles. An inner ring comprises nurse-like populations (NC1) branching from meiotic cyst stages and NC2 and NC3 from follicles. (*B*) Dot plot comparing marker gene expression in indicated clusters. (*C*) Marker gene feature plots on UMAP coordinates. (*D*) Germline cyst development visualized by EdU labeling (1.5 h, magenta), telomere FISH (yellow), and DAPI (cyan). Premeiotic S-phase: telomeres cluster at the nuclear envelope, while “leading” cystocytes (red arrowhead) start bouquet formation. Leptotene–zygotene: telomere bouquet formation and rDNA amplification onset (magenta arrowheads). Pachytene: nuclear size increases, rDNA amplification forms an “rDNA cap.” Early diplotene: cysts fragment to form primordial follicles, telomere dispersal. Stage I follicles: large GV, LBC, telomere detachment.

To investigate early female germline development in *X. laevis*, we performed three replicates of scRNA-seq on postmetamorphic ovaries which are enriched for germ cells undergoing cyst formation and different stages of meiotic prophase I. Transcriptomes were generated from 18,410 cells, including 8,544 germline and 9,866 somatic cells, and processed using Seurat. Germline cells were identified based on expression of conserved markers such as *ddx4* and *dazl*, and subsequently reclustered to resolve transcriptional heterogeneity. UMAP analysis revealed 12 clusters forming a continuous developmental trajectory with two distinct arrangements (Fig. 1*A*): an outer ring (9 clusters), representing the developmental progression from a previously uncharacterized GSC cluster through cyst formation, early meiotic prophase, and early follicle stages, and an inner ring (NC1, NC2, NC3), corresponding to nurse-like cells. Cluster identities were annotated based on stage-specific marker expression (Fig. 1 *B* and *C* and *SI Appendix,* Table S1) and supported by morphological data from EdU labeling and telomere DNA FISH experiments (Fig. 1*D*).

The GSC cluster was positioned at the tip of the germline trajectory, consistent with an undifferentiated state, and specifically expressed *piwil4* and other markers (see the next section). Just downstream of the GSC cluster, cells expressed *cyp26a1*, a meiotic entry repressor (45), consistent with oogonia forming germline cysts. A neighboring G2/M cluster, characterized by high expression of *ccnb3*, *cdk1*, *melk,* and *mki67*, represented actively dividing cells (46). The premeiotic S cluster, marked by *rec8* (47), corresponded to cystocytes undergoing DNA replication, confirmed by EdU labeling and the onset of telomere clustering. Cells expressing *spo11*, *dmc1*, *sycp3*, and *hormad1*—key genes involved in meiotic double-strand break formation, recombination, and synaptonemal complex assembly—were annotated as leptotene–zygotene cysts (48). These cells exhibited prominent telomere bouquets and showed early nuclear polarization, characterized by the repositioning of the nucleolus and the initiation of rDNA amplification. During early pachytene, elevated expression of the same markers and *syce2* coincided with the formation of a prominent rDNA cap opposite the telomeres. The late pachytene cluster, defined by continued expression of *syce2* and *hormad1*, aligned with increasing nuclear size and a fully developed rDNA cap. In the early diplotene stage, rDNA amplification ceased, telomeres began to disperse, and *velo1* was expressed, a marker of the Balbiani body (49). Induction of *figla*, an oocyte-specific transcription factor (50), marked the transition to prefollicular oocytes. Finally, cells expressing *zp4* and *velo1* were annotated as stage 1 follicles (27) with enlarged germinal vesicles (GV) and telomeres detached from the nuclear membrane.

The cells in the NC clusters of the inner ring show low UMI values (Fig. 7*E*) compared to clusters on the outer ring. An early arising group of mouse nurse cells, which also cluster in the similar mouse inner ring, also show low UMI, perhaps due to their early transfer of cytoplasm toward the cyst pro-oocyte (44). *Xenopus* nurse-like cells in cluster NC1 arise early in meiosis and showed many similarities to early mouse nurse cells, for example in reduced expression of the key RNA regulator *daxl* (*SI Appendix*, Fig. S1 and Table S2). The NC1 cluster also downregulated a group of meiosis specific genes: *sycp1, sycp3, syce2, dmc1, spata22*, cilia regulator *arl3*, *hormad1*, and the chromatin regulator *macroh2a2* (*SI Appendix*, Table S2). These same genes are also downregulated in the early mouse NC cluster 19 (44). Overexpressed genes in NC1 include *42sp43* and *42sp50*, a *Xenopus* oocyte specific translational regulatory complex (8-12×), *tmsb15* and *tmsb4x* both thymosin beta proteins implicated in sequestering g-actin, and other genes with potential effects on nurse cell function. In contrast to NC1 downregulated genes, NC1 overexpressed genes were mostly not detected in mouse oogenesis. These findings show that the expression of genes in nurse-like cells is highly regulated, and in some cases changes are conserved from mouse to *Xenopus*.

Multiple genes down and up-regulated in NC2 and NC3 were also examined relative to expression in stage 1 follicles. A sample of upregulated genes included *ddx25* (NC2, NC3), DEAD box helicase, involved in RNA based transcript regulation in the testis, whose function in the ovary is little studied, *rab11fip1* a rab11endosome trafficking modulator, *larp6* a translational regulator, *kif20b* a microtubule motor and meiotic spindle regulator. Downregulated genes include *acp5* (acid phosphatase) whose ovarian function is unknown, *not* (notochord homeobox) and others. In contrast, NC2 and NC3 germ cells express genes characteristic of follicular germ cells. These cells may derive from oocytes of a “wave 1” subpopulation of *Xenopus* follicles that are programmed to turn over, while their somatic cells carry out a specialized function (51).

### Identification and Properties of a *Xenopus* Female GSC Population.

**Fig. 2.**
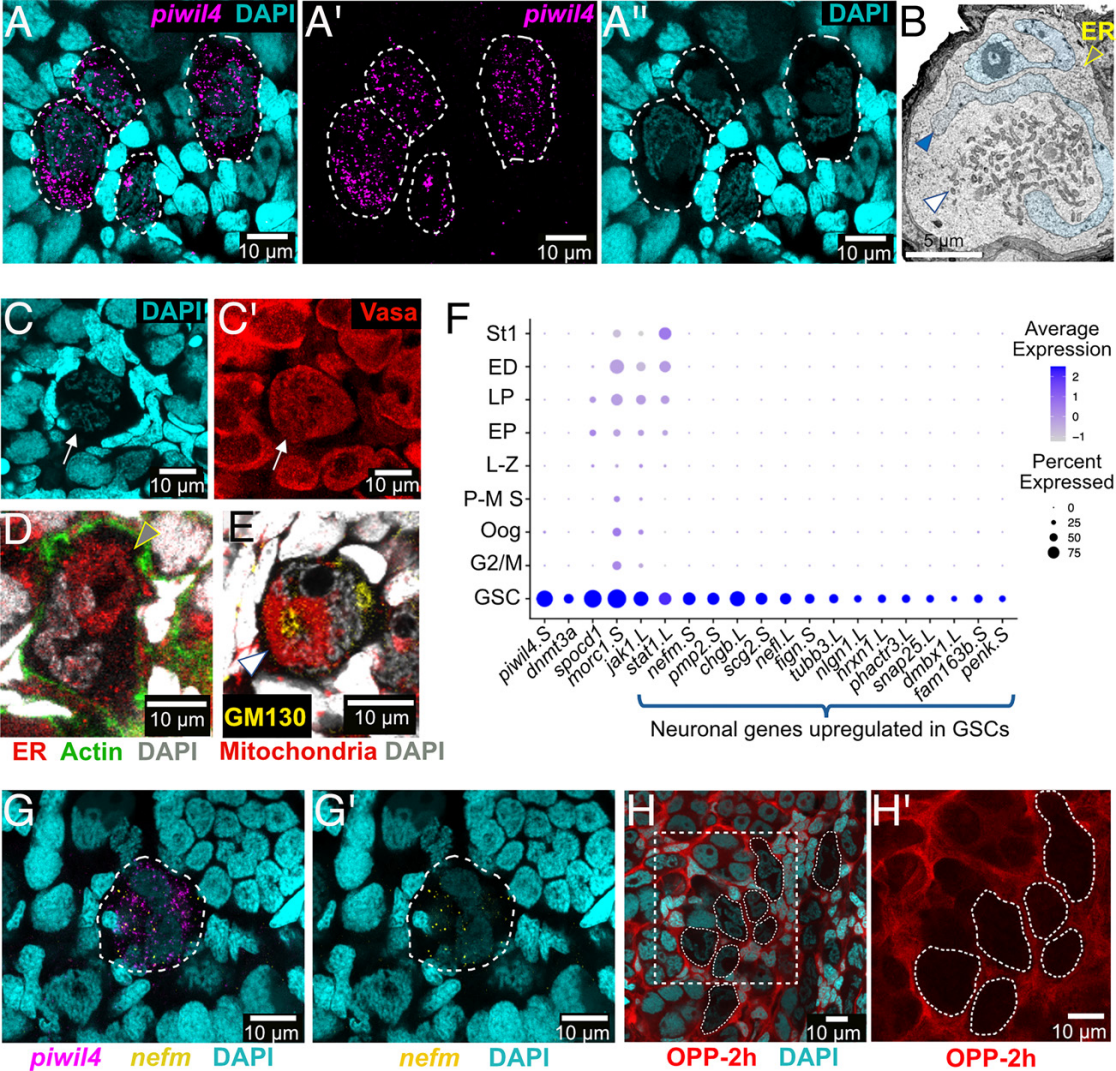
GSC identification. (*A–A”*) Whole-mount juvenile *Xenopus* ovary. HCR RNA-FISH for *piwil4.S* (magenta), DAPI (cyan). (*B*) GSC electron microscopy reveals polymorphic nucleus (blue), mitochondrial cloud (white arrowhead), ER-like structures (yellow arrowhead). (*C* and *C’*) GSC: Vasa (red), DAPI (cyan). (*D*) GSC stained with PDI (red), phalloidin (green), and DAPI (gray). (*E*) GSC stained for mitochondria (citrate synthase, red), Golgi (GM130, yellow), and DAPI (gray). (*F*) Dot plot comparing expression patterns of selected marker genes for GSCs across different germ cell clusters. (*G* and *G’*) Whole-mount juvenile *Xenopus* ovary: two-color HCR RNA FISH for *piwil4.S* (magenta) and *nefm.S* (yellow), DAPI (cyan). (*H* and *H’*) Whole-mount juvenile *Xenopus* ovary: OPP (2 h, red), DAPI (cyan). (*H’*) Magnified rectangle in H: quiescent GSCs: white dashed outlines.

At the origin of the germline trajectory, we identified a transcriptionally distinct cluster defined by high and specific expression of *piwil4*. Using hybridization chain reaction fluorescence in situ hybridization (HCR-FISH) with *piwil4.S* RNA probe (Fig. 2 *A*–*A’’*), we validated these cells as large, *ddx4*-positive (Fig. 2 *C* and *C’*), individual germ cells with distinctive multilobed nuclei preferentially localized near the ovarian surface. This cell type closely resembles the “primary oogonial cells” previously described morphologically in *Xenopus* (25). We also detected similar *piwil4*-expressing cells in the adult ovary, specifically within the ovarian epithelium (*SI Appendix,* Fig. S2). In both juvenile and adult ovaries, these cells exhibit characteristic features of undifferentiated germ cells: Their nuclei are large and polymorphic, with dispersed chromatin as shown by DAPI staining (Fig. 2*A”* and *SI Appendix,* Fig. S2), they display a prominent mitochondrial cloud (Fig. 2 *B* and *E*) localized around the centrosome and an enriched endoplasmic reticulum (ER) cluster (Fig. 2 *B* and *D*), reminiscent of the spectrosome found in Drosophila GSC.

Molecularly, this cluster also expressed *spocd1* (LOC108707506) and *morc1* (Fig. 2*F*), two core effectors of the PIWI-piRNA pathway involved in transposon silencing, previously shown to be critical in mouse gonocytes (52). Their coexpression with *piwil4* suggests that *Xenopus* GSCs initiate transposon repression early in germline development. Additional markers, including *dnmt3a* (LOC108701970, DNA methyltransferase 3a) and *chgb* (chromogranin B), also reported in zebrafish GSCs (39), were enriched in this cluster (Fig. 2*F*). In this cluster, we also observed upregulation of the JAK1-STAT1 signaling pathway known for its conserved role in stem cell regulation across different species (53) (Fig. 2*F*).

Unexpectedly, the cluster showed strong enrichment for genes typically associated with neuronal function (Fig. 2*F* and *SI Appendix,* Fig. S3). These included cytoskeletal and synaptic components (*neurofilament medium and light chains, βIII-tubulin, neuroligin 1,* and *neurexin 1*), neuropeptide precursors and secretory factors (*VGF, secretogranin II, proenkephalin, chromogranin, synuclein beta, neuropeptide y*, and *synaptosome-associated protein*), and regulators of neuronal identity (*fam163* and *diencephalon/mesencephalon homeobox)* (Fig. 2*F* and *SI Appendix,* Fig. S3). Coexpression of *piwil4* and *nefm* was confirmed by two-color HCR-FISH (Fig. 2 *G* and *G’*).

In line with their stem status, GSCs also appeared to be translationally quiescent compared to downstream germ cells. This was evident from low O-propargyl-puromycin (OPP) incorporation (Fig. 2 *H* and *H’*), indicating reduced global protein synthesis, and was supported by scRNA-seq data, where different ribosomal protein genes were significantly downregulated in the GSC cluster (*SI Appendix*, Fig. S3*A*). Such translational repression is a hallmark of many stem cell populations and has been implicated in preserving genome integrity and stemness (54).

To test whether adult *Xenopus* can utilize GSCs to replenish the oocyte pool, we examined adult females several years after partial ovariectomy. We observed small, regenerated ovarian lobes (*SI Appendix*, Fig. S2 *C*–*C”*) that contained germline cysts at various stages, including dividing oogonia, meiotic cysts, and stage I–II follicles, as well as cells with features consistent with GSCs. In contrast, such structures were not found in frogs that had not undergone ovariectomy. These findings suggest that the adult *Xenopus* ovary retains regenerative capacity and harbors a GSC population capable of reinitiating oogenesis following tissue loss.

### Developing Cysts Are Stably Interconnected by a Microtubule-Rich FLS.

**Fig. 3.**
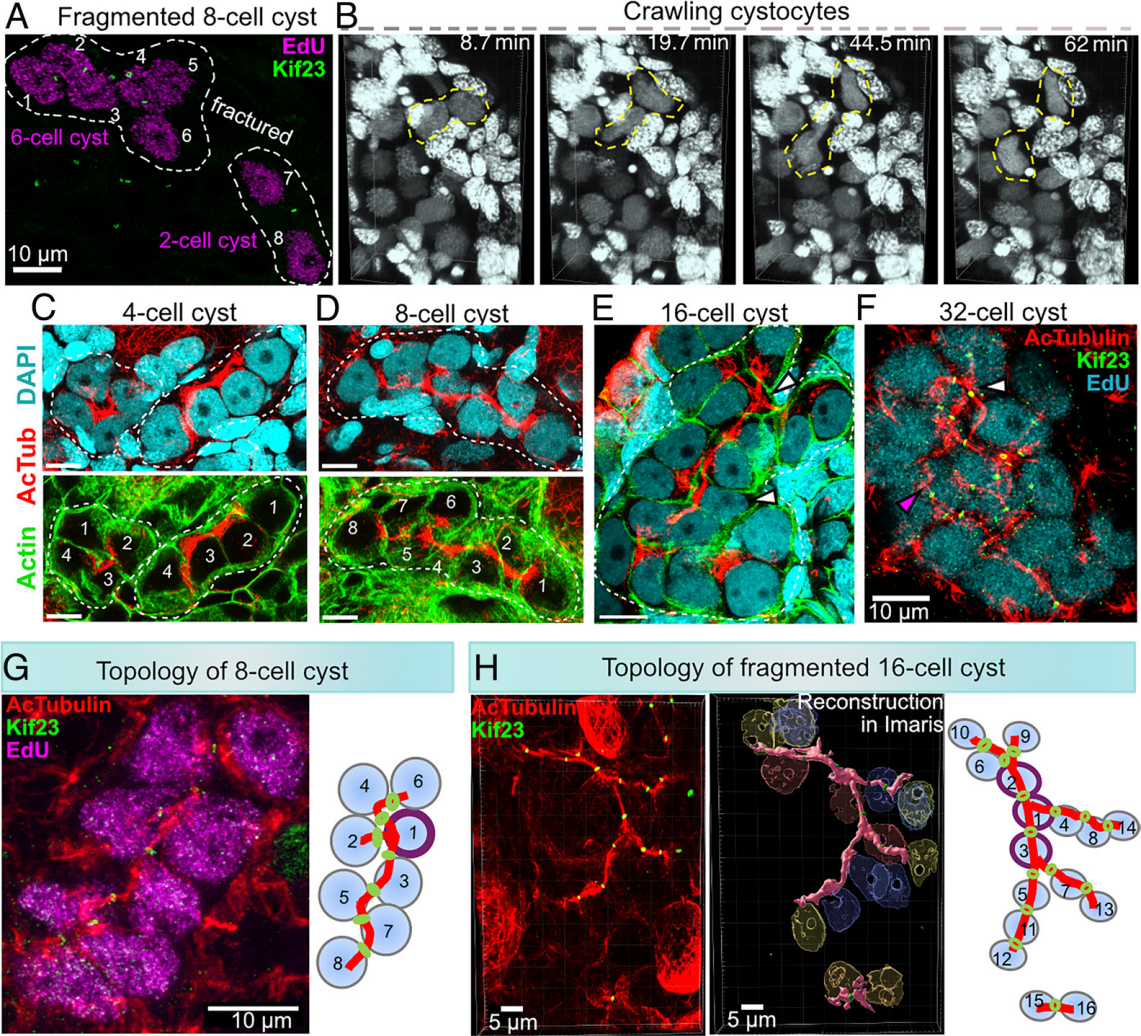
FLS within germline cysts. (*A*) Germline cyst: EdU (~2 h, magenta), Kif23 (ring canals, green) showing fragmentation into a 6-cell group and 2-cell group. (*B*) Juvenile ovary imaged live (~2 h) showing motile cystocytes (yellow dashes), Hoechst (gray). (*C–E*) 4-,8-16-cell cysts: acetylated microtubules (red), phalloidin (green), DAPI (cyan). (Scale bar, 10 µm.) (*F*) 32-cell cyst: EdU (cyan), acetylated microtubules (red), Kif23 (green). (*G*) 8-cell cyst topology: EdU (magenta), Kif23 (green), acetylated microtubules (red); cartoon: deduced connectivity. (*H*) Fragmented 16-cell cyst (14-cells, 2-cells): Kif23 (green), acetylated microtubules (red); Imaris 3D-reconstruction showing cyst nuclei, FLS (pink); cartoon: deduced interconnections.

Downstream of the GSC population, germline cysts begin to form through mitotic divisions of oogonia and early meiotic entry, primarily along the ovarian periphery (26, 32). To identify and study developing cysts, ovaries from 50- to 60-d-old froglets were labeled with EdU and analyzed as whole mounts. Using 3D reconstruction in Imaris, we identified clusters of 2-, 4-, 8-, 16-, and 32 EdU-positive cells as cysts (*SI Appendix*, Fig. S4 *A* and *B*). However, as many as ~17% of cysts did not contain 2^n^ cells, such as 6-, 19-, or 25 cells (*SI Appendix,* Fig. S4 *A* and *B*), suggesting that cyst fragmentation might occur, similar to observations in mice (55, 56). An example of such a cyst fortuitously labeled with EdU and ring canals (via Kif23, a kinesin family member and component of the centralspindlin complex) as an 8-cell cyst (Fig. 3*A*) now shows two closely associated but separate 6-cell and 2-cell groups with no ring canal between them. Live imaging of dissected ovaries stained with Hoechst revealed that cystocytes exhibit active crawling-like movement which may contribute to cyst breakage due to stretching (Fig. 3*B*, *SI Appendix,* Fig. S4*C*, and Movie S1). This type of germline cell behavior was observed especially during oogonial stages before meiotic prophase. In addition, unlike the perfect synchrony observed in Drosophila germ cell cysts, *Xenopus* cysts showed limited but detectable asymmetry in cell cycle progression with distinguishable “leader” and follower cells as observed in both fixed and live samples (*SI Appendix*, Fig. S4 *D* and *E* and Movie S2). It is possible that the same leader cell also advances first through the meiotic stages within the cyst, as suggested by telomere FISH results in which only one cell in the cyst displayed clustered telomeres while the others had not yet (Fig. 1*D*, the red arrowhead points at leader cell).

A major feature of Drosophila cysts is the fusome, an asymmetric, microtubule-rich organelle containing smooth ER cisternae and more than 20 identified proteins (8). Immunostaining of ovaries for acetylated tubulin, actin, and DAPI revealed that bundles of stable microtubules (MT) traverse the sister cells in a branched path within 4-, 8-, and 16-cell cysts (Fig. 3 *C*–*E*). These MT bundles pass through intercellular bridges (IBs) (ring canals) as shown in a 32-cell cyst labeled for Kif23, a kinesin-like protein 23 (Fig. 3*F*). Interestingly, microtubules are unevenly distributed within the cyst: Cells with multiple intercellular connections often exhibit thicker microtubule bundles, suggesting some sort of polarized organization of the cyst (Fig. 3 *E* and *F*; white arrowheads indicate cells with 3 or 4 ring canals and prominent microtubule bundles; the pink arrowhead marks a cell with thinner bundles). Due to their resemblance to the Drosophila fusome, we refer to these structures as the “FLS.” Like the fusome, FLS exhibits branching, although *Xenopus* cysts show less branching than maximum branching feature of Drosophila cysts. Due to the compact architecture of *Xenopus* cysts, full topological reconstruction is rarely possible. Nonetheless, in well-preserved examples, we were able to analyze cyst organization and identify key differences from Drosophila. In 8-cell cysts, only one cell usually had three ring canals, compared to two such cells in fly cysts (Fig. 3*G*). In a fragmented 16-cell cyst, we observed a split into 14- and 2-cell groups, and within the 14-cell portion, three cells each had three ring canals, unlike the Drosophila pattern of two cells with four connections (Fig. 3*H*). These findings suggest that while *Xenopus* cysts share overall architecture with insect cysts, their topology and fragmentation dynamics are distinct. Notably, the FLS did not stain with spectrin, actin, or the Drosophila hts antibody (1B1), indicating possible molecular divergence despite morphological similarity.

### Germline Cysts and Their FLS during Mitotic and Early Meiotic Stages.

**Fig. 4.**
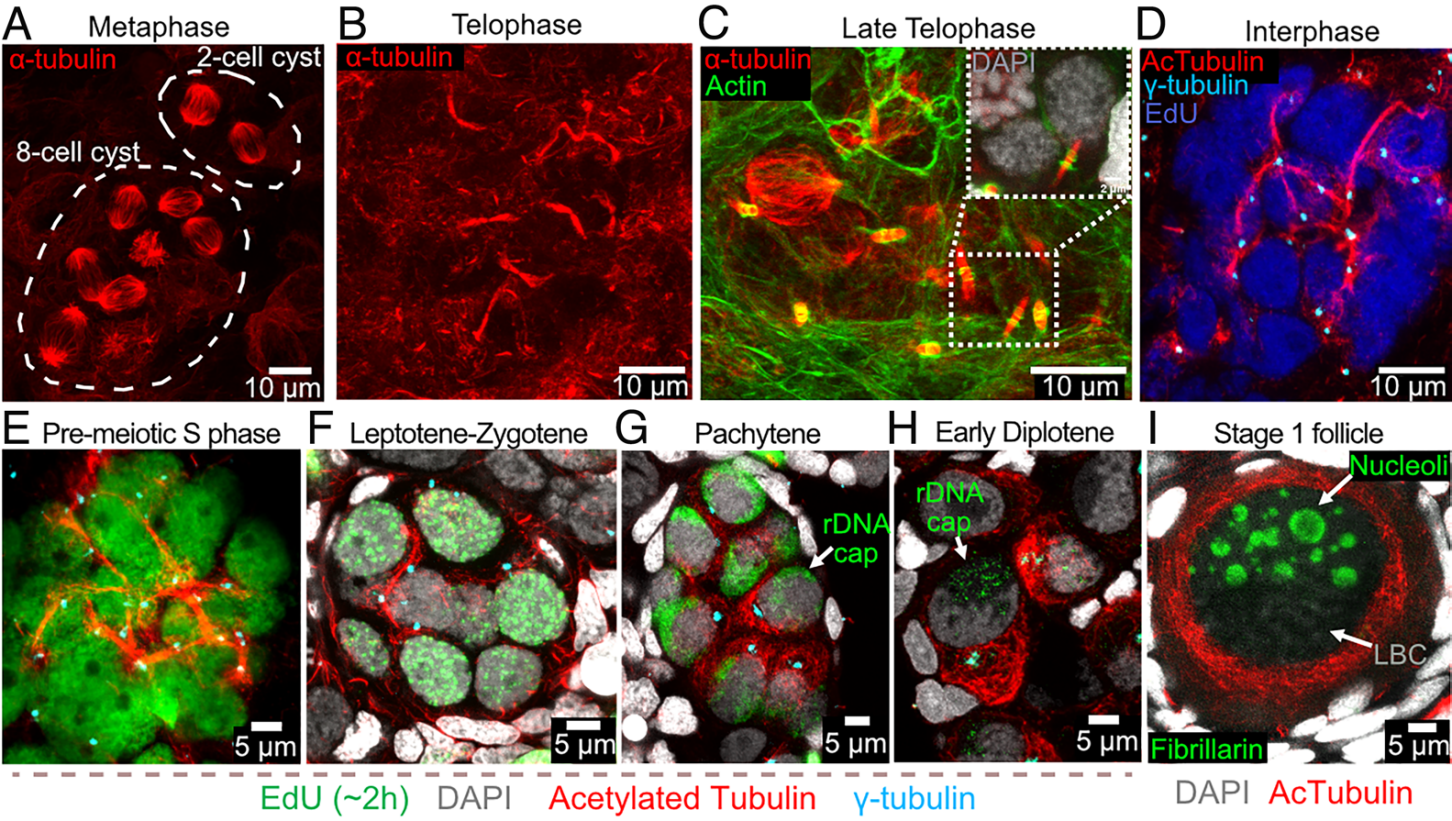
FLS during germline cyst development. (*A*) Metaphase germline cysts (8-cell, 2-cell): α-tubulin (red). (*B*) 16-cell cyst during late telophase: α-tubulin (red) reveals 8 midbodies. (*C*) 8-cell cyst: midbodies elongating to span IBs without gaps. The dashed box is magnified in the upper right corner to show 2 cells (DAPI, gray) interconnecting by IB (green) and MT (red). (*D*) During interphase, oogonial cysts form FLS interconnecting all sister cells via microtubules: EdU (blue), acetylated microtubules (red), γ-tubulin (centrioles, cyan). (*E–H*) Juvenile ovaries: acetylated microtubules (red), γ-tubulin (cyan), EdU (green), DAPI (gray). Maximum projections of selected optical slices. (*E*) Premeiotic S-phase cyst: EdU (green), acetylated MT (red), shows FLS microtubules extending from MTOCs (γ-tubulin, cyan). (*F*) Reduced FLS structure in leptotene–zygotene cyst. (*G*) Pachytene-stage cyst with further reduced FLS. (*H*) Diplotene primordial follicle: MTs accumulating (vegetal pole) near forming Bb. (*I*) Stage I follicle with multiple nucleoli (fibrillarin, green) and forming LBC: acetylated MTs are enriched around GV.

The fusome in well-studied systems arises from the metaphase spindles following cystocyte mitotic division, whose turnover is arrested by the failure to complete cytokinesis (57–59). In forming *Xenopus* cysts, spindles appeared normal at metaphase (Fig. 4*A*). In late telophase, spindle microtubules contract and bundle within the intercellular bridge between two sister cells, forming the midbody (Fig. 4*B*). Subsequently, these midbodies within the cyst become stabilized, giving rise to stable IBs with no microtubule gap within bridges (Fig. 4*C*). Exactly how this stabilized midbody leads to a permanent intercellular bridge and altered microtubule polarity is not well understood. As the cyst enters interphase, microtubule bundles interconnect all cystocytes, forming a network in which stable microtubules contribute to cystocyte connectivity (Fig. 4*D*). We used EdU labeling (1 to 6 h) to identify cysts and determine the cell cycle stage of the cells within them. While all EdU-positive cysts (S-phase) show strong microtubule interconnections (*SI Appendix*, Fig. S5 and Movies S3–S6), many EdU-negative cysts with the same morphology (interphase) also maintain robust microtubule interconnections.

Early meiotic prophase occurs within vertebrate and invertebrate germline cysts suggesting that cyst formation evolved as an animal prelude to meiotic entry. In Drosophila, stage-specific changes in the fusome have long been noted during meiosis (10). Consequently, we next studied the dynamics of *Xenopus* FLS during early meiotic prophase (Fig. 4 *E*–*I* and *SI Appendix*, Fig. S6). Fig. 4 *E*–*I* summarizes changes in the morphology of the FLS during an approximately 30-d developmental period between premeiotic S phase and diplotene follicles where the cyst has broken down. As in premeiotic cysts (Fig. 4*E*) the FLS in leptotene–zygotene (Fig. 4*F*) continue to span cyst cells, and associate with centrioles (γ-tubulin). However, the bundles connecting cells become less compact and spread more widely in the cell during pachytene (Fig. 4*G*). During early diplotene (Fig. 4 *H* and *I*), cysts split into individual follicles, where MTs have spread through much of the cytoplasm.

### Transport of Vesicles and Materials between Cyst Cells via Ring Canals.

**Fig. 5.**
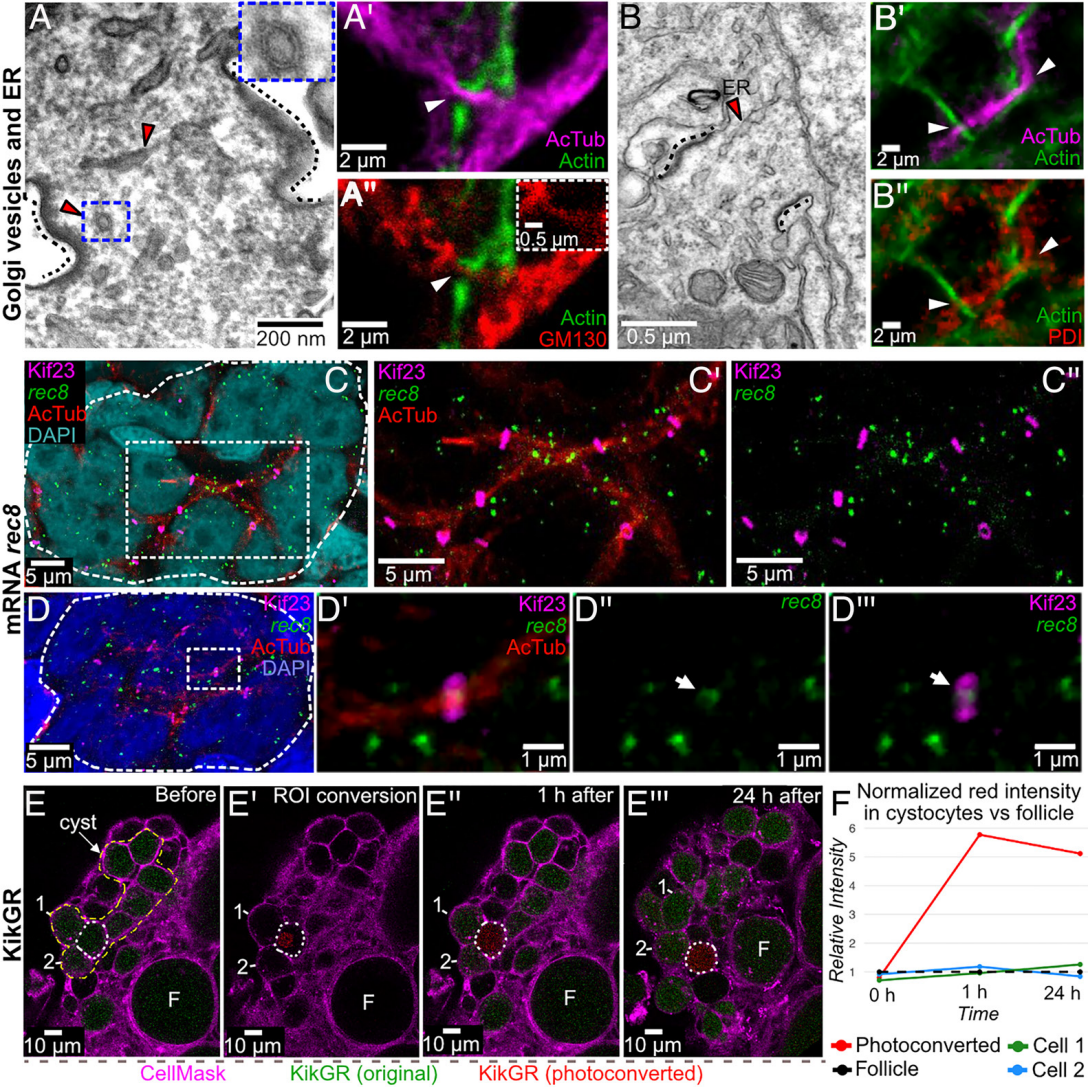
Vesicles, ER, and mRNA, but not photoconvertible KikGR, move through ring canals. (*A–A’’*) Golgi-derived vesicles within IBs. (*A*) EM of IB (black dashed line) shows multiple vesicles (red arrowheads) within its lumen. The blue dashed box shows a high-magnification image of the vesicle in the upper-right corner. (*A’* and *A’’*) Whole-mount ovary: acetylated tubulin (magenta), phalloidin (green), GM130 (red) reveals Golgi-derived vesicles localized along microtubules (arrowheads). (*A’*) The dashed box highlights the region at the arrow tip showing Golgi-derived vesicles in the IB. (*B–B’’*) ER-like structures within IBs. (*B*) EM shows ER-like structures (red arrowhead) within IB lumen (black dashed line). (*B’* and *B’’*) Whole mount ovary: acetylated tubulin (magenta), phalloidin (green), PDI (red) shows ER colocalization with microtubules crossing IBs (arrowheads). (*C–C’’*) HCR RNA-FISH with a *rec8* probe (green), acetylated tubulin (red), ring canals (Kif23, magenta), and DAPI (cyan). (*C’* and *C’’*) Boxed region in *C*: showing *rec8* mRNA enriched on microtubules in a cystocyte with four ring canals. (*D–D’’’*) HCR RNA-FISH with *rec8* probe (green), acetylated tubulin (red), ring canals (Kif23, magenta), DAPI (cyan). (*D’–D’’’*) Boxed region in *D* showing *rec8* RNA molecule located within ring canal lumen. (*E–E’’’*) Photoconversion of KikGR (green to red) within a single cyst cell. CellMask (cell membranes, magenta). Converted cell (white dashes). (*E*) Before conversion. (*E’*) ROI showing single cell conversion. (*E’’*) 1 h postconversion. (*E’’’*) 24 h postconversion. (*F*) Red signal intensity in neighboring cystocytes vs. an unconnected follicle (F). Y-axis: relative red intensity; X-axis: time. Sister cells 1 and 2 correspond to those in (*E–E’’’*); F (follicle) used for normalization.

We carried out electron microscopy on *Xenopus* ovaries and analyzed serial ultrathin sections to identify intercellular transport by visualizing organelles within the lumen of ring canals (Fig. 5 *A* and *B* and *SI Appendix,* Fig. S7). Electron microscopy revealed that IBs between cyst cells were invariably filled with cytoplasmic material, including Golgi vesicle-like (Fig. 5*A*) and ER-like structures (Fig. 5*B*). Similar vesicle-like contents were recently reported in zebrafish cysts (60). Immunostaining for GM130 (Golgi matrix protein) in whole ovaries detected vesicle-like structures colocalized with microtubules crossing the bridge (Fig. 5 *A’* and *A”*). Similarly, immunostaining for PDI (protein disulfide isomerase) revealed ER structures associated with acetylated microtubules in bridges between oogonia cystocytes (Fig. 5 *B’* and *B”*).

We also examined how various organelles change in a cyst during meiotic stages (*SI Appendix*, Fig. S8). During oogonial stages, ER can be found colocalized with microtubule bundles. ER appears enriched in a subregion of pachytene cyst cells that contains the centrosome and Golgi. In the early diplotene stage shown, the cyst has broken down entirely and has formed a new primordial follicle with ER enrichment in the forming Bb. Golgi and γ-tubulin staining shows that centrosomes and Golgi are also located asymmetrically in the cell (*SI Appendix*, Fig. S8*B*). In Drosophila, centrioles migrate through IBs along the fusome toward the pro-oocyte in late pachytene. In mouse nurse cells, centrosomes, mitochondria, Golgi, and cytoplasm pass through membrane gaps into the pro-oocyte (43). To investigate whether directional organelle transfer occurs in *Xenopus*, we stained centrioles using γ-tubulin and centrin-2 (*SI Appendix,* Fig. S8 *C* and *D*). γ-Tubulin foci expanded at the pachytene stage and declined at the stage 1 follicle. In contrast, centrin-2 consistently marked two distinct dots per cystocyte, indicating that each cystocyte retains its own centrioles. No evidence of centriole transfer was observed based on constancy in the number of centrin-2 foci within individual cells. Both electron microscopy and immunofluorescence failed to detect mitochondrial passage through *Xenopus* IBs. In contrast to *Drosophila*, but similar to mice, ring canal diameters in *Xenopus* remained relatively constant across all stages examined (*SI Appendix,* Fig. S9 and Movie S7).

To test whether RNA passes through ring canals, we performed HCR-FISH for *rec8*, a marker of premeiotic S phase, combined with immunostaining for Kif23 and acetylated tubulin. *Rec8* mRNA localized along microtubule bundles, particularly in cystocytes with multiple ring canals (Fig. 5 *C*–*C’’*), and was occasionally detected within the center of ring canal lumens (Fig. 5 *D*–*D’’’*), suggesting mRNA transport between cyst cells. Whether this transport is directional or selective for specific transcripts, such as oskar mRNA in Drosophila, remains an open question. In support of selective sharing, recent work in mouse testes showed that certain mRNAs are always shared between sister cells in cyst (e.g., *Sycp3*), others are only partially shared (e.g., *Ccdc28a*), and some are never shared (e.g., *Smok2b*) (61).

To test whether cystocytes exchange cytoplasmic proteins, we performed live imaging of ovaries from transgenic frogs (*Xla.Tg(CAG:KikGR*). The KikGR (green) protein undergoes irreversible conversion to red fluorescent protein following photoconversion using UV light. Isolated ovaries were stained with CellMask™ plasma membrane dye, and KikGR protein was photoconverted from green to red in a single cystocyte using UV light. Imaging before and after photoconversion (1 h and 24 h postconversion) showed no detectable transfer of labeled proteins between cystocytes (Fig. 5 *E*–*E’’’* and *F*). Neither green KikGR protein entered the photoconverted cell, nor did red KikGR protein diffuse to neighboring cystocytes. As a positive control for protein mobility, we photoconverted a small region of cytoplasm in a stage 1 follicle; in this case, red KikGR rapidly spread throughout the entire follicle within milliseconds, confirming that the lack of exchange in cysts was not due to limitations of the imaging or photoconversion system. These findings suggest that IBs in *Xenopus* do not facilitate significant KikGR protein sharing over a 24-h period.

### Microtubule Connections between Cystocytes Can Reform Following Disruption.

**Fig. 6.**
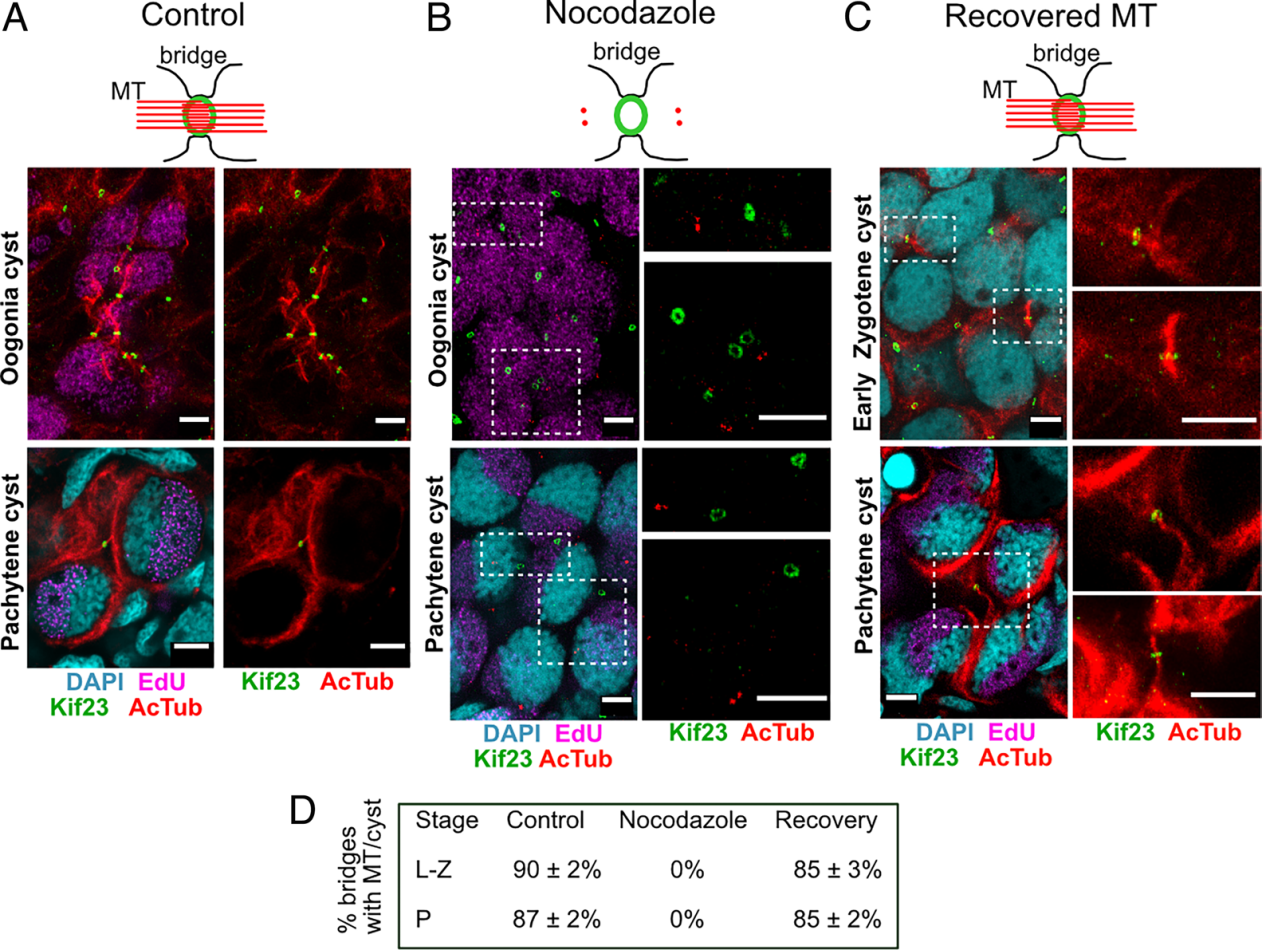
Microtubule interconnections between cystocytes are actively maintained. Juvenile ovaries showing microtubule networks in oogonial and early meiotic cysts. (*A*) Control, DMSO-treated. (*B*) 0 h after nocodazole treatment—microtubules are reduced to centrioles, ring canals remain intact. Two boxed regions on the left are magnified in the panels on the right. (*C*) MT regrowth after nocodazole washout. The bottom-right panel in (*C*) shows another example of MT recovery in the IB. (*D*) Quantitation: bridges with microtubules (%) in control and recovery. Acetylated tubulin (red), Kif23 (green), EdU (1 h before recovery, magenta), DAPI (cyan). (Scale bar, 5 µm.)

To assess the role of microtubule connections in cyst development and intercellular exchange, we examined their recovery following complete depolymerization. While spindle remnants likely initiate intercellular microtubule formation, we tested whether microtubules could reform independently of midbody-derived structures. To test this, we treated ovaries with Nocodazole, which completely depolymerized acetylated microtubules in germline cysts within 15 to 20 h (Fig. 6 *A* and *B*). Notably, in the whole ovary only cells with features of GSCs retained acetylated microtubules even after 20 h of nocodazole treatment (*SI Appendix,* Fig. S10*A*). Importantly, ring canals remained unaffected (Fig. 6*B*), and the vast majority of cysts appeared healthy, although some showed signs of degradation. Following drug washout, cytoplasmic microtubules fully recovered within 10 to 24 h. Notably, we also observed microtubule recovery within IBs at all germline cyst stages (Fig. 6*C*), suggesting that microtubules can regrow within bridges independently of spindle remnants. To avoid the confounding effect of new spindle formation in dividing oogonial cysts during recovery, we focused our analysis on meiotic-stage cysts, which progress more slowly and are unlikely to divide within the recovery time frame. Quantification revealed that in control samples, approximately 85 to 92% of analyzed IBs in meiotic cysts at leptotene–zygotene and pachytene stages contained microtubules. Following nocodazole washout, a similar percentage of bridges showed microtubule recovery and reconnection (Fig. 6*D*). Interestingly, after microtubule recovery, particularly in pachytene-stage cysts, we frequently observed long MT bundles, up to 20 to 30 μm in length, spanning between sister cells that had moved apart but remained connected by intercellular bridge (*SI Appendix,* Fig. S10*B*). Such configuration was never seen in untreated controls, suggesting that in the absence of MTs, cyst cells may drift apart without fully disconnecting.

### *Xenopus* Cysts Contain Nurse Cells That Turn Over and Do Not Form Oocytes.

Previously, all individual cystocytes were proposed to survive and differentiate into oocytes, based on a dearth of TUNEL-positive nuclei observed in the postmetamorphic ovary (32). Subsequently, studies in Drosophila (62), and later in the mouse (44) showed that apoptosis is not used routinely for nurse cell turnover by these species. Instead, a process of programmed cell death involving acidification by somatic cells and degradation near other cyst cells takes place (62, 63). We developed a lineage-tracing method using EdU pulse–chase labeling, since a Cre-loxP genetic system is not available for *Xenopus* to revisit this issue.

We injected EdU into 125 metamorphic *Xenopus* tadpoles and dissected their gonads every 2 d to track cystocyte fate. This approach enabled us to establish the timeline of cyst development, tracing the progression from oogonial stages through early meiotic prophase (leptotene, zygotene, and pachytene) to the formation of individual follicles. Oogonial cysts appeared up to 4 d after the EdU pulse, leptotene–zygotene cysts up to 9 d, pachytene cysts up to 15 d, early diplotene cysts up to 27 d, and stage I follicles after 27 d (*SI Appendix,* Fig. S11). These findings suggest that, on average, cystocytes require approximately 1 mo to progress from oogonial divisions and early meiotic prophase to the formation of individualized stage 1 follicles which coincides with a conclusion from Coggins and Gall (26).

**Fig. 7.**
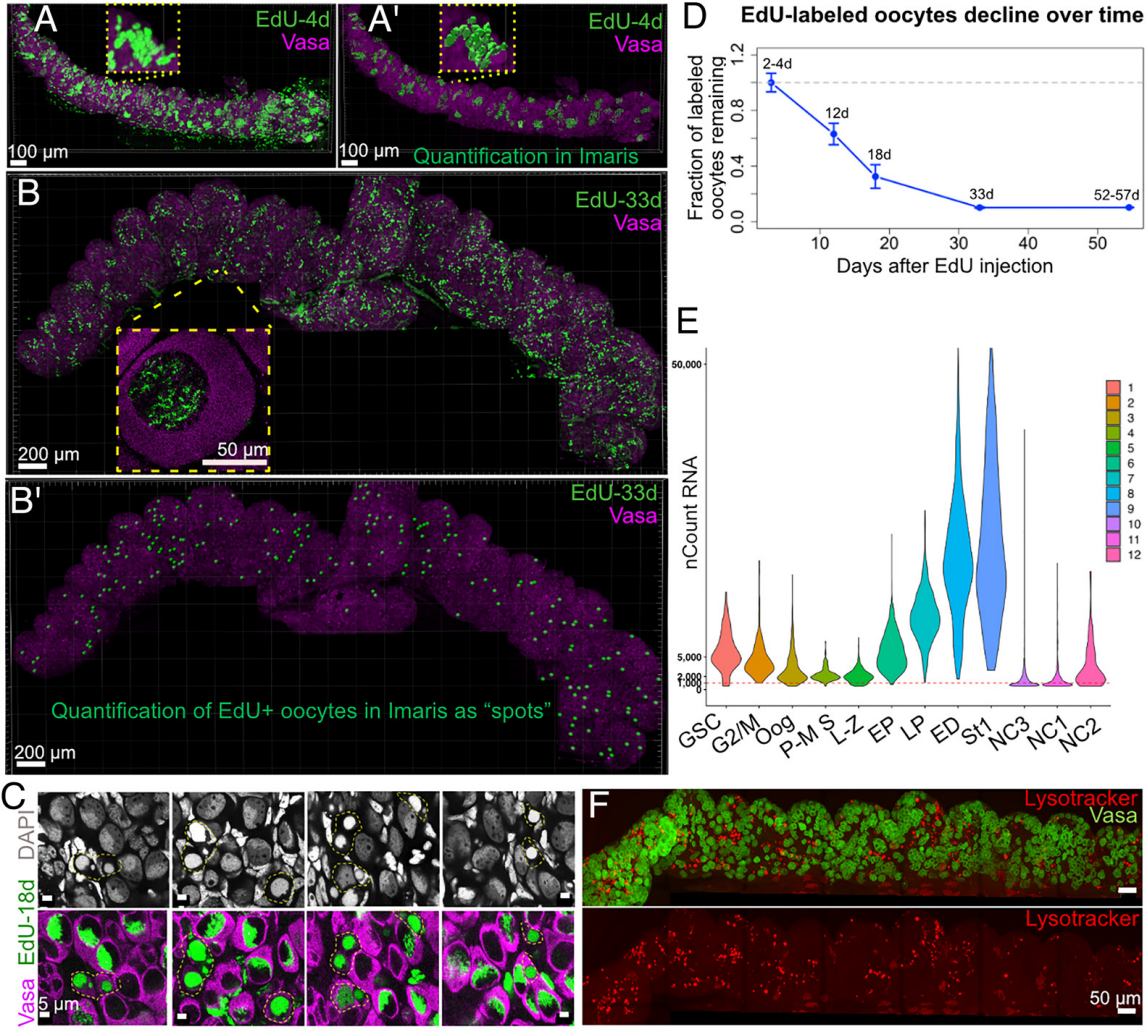
Cysts contain mostly nurse cells that turn over. (*A* and *A’*) Juvenile ovary 4 d after EdU injection. Germline cells (Vasa, magenta), EdU (green). (*A’*) Manual quantification (Imaris: “Spot,” “Cell” functions). Boxes indicate germline cyst from (*A*) reconstructed (*A’*). (*B* and *B’*) Analysis as in (*A*), 33 d after EdU injection. (*C*) 18 d post–EdU injection. Dying EdU-positive (green) germline cells (dashed regions) in late pachytene/early diplotene cysts, (Vasa, magenta), (DAPI, gray). (*D*) Declining EdU-labeled oocyte numbers 2 to 57 d after EdU pulse. (*E*) Distribution: nCount_RNA (UMI counts) per cell in indicated germline clusters. (*F*) Extensive programmed cell death of germ cells visualized by acidified nuclear remnants (Lysotracker, red), (Vasa, green).

Because our labeling method only marks cysts undergoing S-phase at the time of EdU exposure, it allows a straightforward situation where cell turnover can be followed over subsequent days with comparative quantification of developmental stages (Fig. 7 *A*–*A’*, *B*–*B’*, and *D* and *SI Appendix,* Fig. S12). It is clear that over this 30 d period, the great majority of cell labeling is lost. We estimated from sampling that there were at least 1,296 germ cells labeled 4d after the EdU pulse. At 33 d, the remaining signal originates from compact somatic nuclei dispersed throughout the ovary, whereas germ cells at this stage are organized into early follicles with developing LBCs, which are only visible upon zooming in (Fig. 7*B*, rectangle box). Moreover, it was easy to count the 217 EdU-labeled cells remaining at 33 d, which microscopic examination verified were all no longer cysts, but young ovarian follicles (Fig. 7*B*, rectangle box; Fig. 7*D*). Thus, it is clear that the great majority of cyst cells (~80%) do not survive to become oocytes (Fig. 7*D*).

Since we examined samples at several intermediate time points, we observed that relatively little turnover took place early, but significant amounts of EdU-positive germ cell death began around 18 d. At this time, most EdU-labeled cystocytes were in late pachytene-early diplotene stages, and many EdU-positive germ cells nearby exhibited morphological features consistent with cell death (Fig. 7*C*). These included a smooth nucleus lacking the characteristic chromosomal bouquet and rDNA cap, as well as a shrunken, Vasa-positive cytoplasm. Such a phenotype of germ cell death was also observed in ovaries without EdU pulse–chase which proves that it is not an artifact of cell death due to EdU incorporation.

Moreover, scRNAseq data revealed clusters in the inner ring with relatively low RNA counts per cell (UMI) (Figs. 1*A* and 7*E*). Cluster NC1 is positioned near leptotene/zygotene cells and near early diplotene cells when the cyst tends to break down. Cluster NC3 is close to the stage 1 follicles and might represent dying germ cells from wave 1 follicles (44, 51). NC1 accounts for 10% of total germ cells, while all 3 NC clusters make up about 20%.

To investigate whether these cells turnover through programmed cell death via acidification, as shown for NC in flies (62, 63) and mice (44), we performed Lysotracker staining on *Xenopus* ovaries. Our results showed that the majority of germ cell nuclear remnants were Lysotracker positive (Fig. 7*F*), consistent with *Xenopus* nurse cell turnover by programmed cell death and with previous observations of low apoptotic cell death in the *Xenopus* ovary (32).

## Discussion

### Transcriptional Identity of GSCs in *Xenopus*.

Our single-cell RNA sequencing atlas of *Xenopus* early germline development provides a comprehensive transcriptional roadmap spanning quiescent GSCs, mitotically dividing cysts, early meiotic cells, and primordial follicles. We were able to capture transcriptomes of rare GSCs and validate their identity by *piwil4* expression using RNA FISH. These GSCs exhibit a distinct transcriptional profile characterized by hallmark features of germline stemness: globally low translation (54), activation of JAK1/STAT1 signaling (53), expression of transposon-silencing effectors (*piwil4, spocd1, morc1,* and *dnmt3a*) (52, 64), and expression of neural genes (*chgb, scg2,* and *npy*).

The selective expression in *Xenopus* female GSCs of genes more commonly associated with neural cells suggests mechanisms worth investigating further. Germ and neural cells are known to share related mechanisms of compartmentalized translational regulation (65, 66) and regulated mRNA and organelle movement on their well-developed microtubule cytoskeletons. Some of these mechanisms might be exclusively used in GSCs, consistent with the unusual GSC cellular structure we observed. Additionally, all animals must dynamically adjust ovarian activity to external circumstances by modulating hormone production via the hypothalamus–pituitary–gonadal axis (HPG), and by enervating particular ovarian tissue, for example to control ovulation. Some of the neural genes expressed in *Xenopus* GSCs such as granin-family proteins encoded by *chgb*, *scg2*, etc. facilitate secretion from neural and other cells. They are highly expressed in the pituitary and adrenal cortex (67), but knockouts of these genes are not essential for fertility. GSCs may secrete signals downstream from systemic inputs, to locally communicate with other ovarian cells and to provide feedback to the HPG axis. All GSC-expressed genes, and especially those encoding proteins currently associated only with neural function require functional validation in GSCs.

Notably, *Xenopus* GSCs express *tubb3* (class III β-tubulin), an isoform previously reported only in terminally differentiated neurons (68) and Sertoli cells (69). In those contexts, *tubb3* is regulated by androgens, which influence cytoskeletal gene expression and promote resistance to cellular stress (69, 70). Strikingly, *Xenopus* GSCs were the only ovarian cell type that maintained highly stable, acetylated microtubules under nocodazole treatment, suggesting enhanced microtubule resilience. These findings raise the possibility that hormonal signals—perhaps androgen-like—may contribute to cytoskeletal stability and GSC maintenance in amphibians.

The fact that adult *Xenopus* females can be induced to ovulate multiple times per year (71) and are capable of regenerating new ovarian lobes after partial ovariectomy, supports the conclusion that *Xenopus* GSCs are not merely rudimentary remnants but are functionally competent stem cells with the capacity to generate new cysts and follicles throughout adult life.

### *Xenopus* Oocytes Arise in Germline Cysts with a Prominent FLS.

*Xenopus* ovarian cysts arise with many of the features that have been described previously in male gametogenesis and most of the characteristics of female premeiotic cells from a wide diversity of animal groups. These include mitotic synchrony, incomplete cytokinesis, formation of a stabilized intercellular bridge, assembly of a microtubule-rich fusome from spindle remnants, readjustment of bridges toward each other in a rosette pattern, and asymmetric accumulation of fusome microtubules and associated materials preferentially in cyst cells containing multiple branches. Two kinds of cells derive from *Xenopus* cysts, a relatively small number of oocytes, and a large number, 80% of more, of nurse cells defined as cyst cells that are programmed to serve functions other than that of oocytes. No longer can lower vertebrates be considered an exception to the evolutionary conservation represented by the cyst program.

A key feature of the *Xenopus* FLS is the exceptional strength and stability of its microtubule-rich connections. These MTs span cystocytes throughout interphase, peak during premeiotic S-phase, and gradually disperse as meiosis progresses—mirroring the developmental pattern of Drosophila fusomes (10). Our observations suggest that this structural stability may be essential for maintaining cyst architecture and coordinated development. Importantly, the FLS demonstrated remarkable plasticity: When microtubules were experimentally depolymerized, cystocytes could drift apart from each other yet remained tethered by ring canals. Upon recovery, long microtubule bundles regrew, spanning even distant cells. Our experiments support the idea that microtubule connections within a cyst are essential not only for maintaining mechanical cohesion and cyst conformation but also for enabling molecular sharing among cystocytes.

Although rare, we observed cyst fragmentation during oogonial stages. Live imaging and fixed material revealed that oogonia exhibit increased motility compared to meiotic cysts, as also recently demonstrated in the mouse (56). This motility likely imposes mechanical stress on IBs. As far as we can tell, fragmentation tends to occur at the cyst periphery, where fusome microtubules are weaker due to their asymmetric distribution. This suggests that the FLS may play a role in mechanically stabilizing cysts and protecting IBs from rupture during periods of increased cell motility.

### Future Nurse Cells Downregulate Key Meiosis Genes Early.

Nurse cells have very different tasks than oocytes, and it has long been known in Drosophila that only the future oocyte and one nurse cell form a synaptonemal complex and prepare for recombination (72). Analyzing gene changes in NC1 nurse cells shows that a similar process takes place in *Xenopus* cysts. Once specified, nurse cells have nothing to gain by continuing with synapsis and recombination, since their genome will never be used in a gamete. Instead, the true oocyte stands to benefit by ensuring that supportive nurse cell transcription will not be adversely affected by ongoing recombination, replication, DNA repair, and meiotic checkpoint activation that could reduce transcriptional output and inactivate chromatin in some chromosome regions.

We examined the earliest *Xenopus* nurse-like cells, NC1, which appear in our scRNA-seq profile at early leptotene. NC1 nurse-like cells express a suite of genes that can be understood as a program for cells switching to a nurse cell state from an earlier cyst cell that retained oocyte potential. They downregulated genes controlling some chromosomal aspects of meiosis, including *sycp1*, *hormad1*, and *dmc1* all of which are required for homologous chromosome synapsis in oocytes and mutate to sterility. Synapsis in turn is essential for recombination and chiasma formation. *Hormad1* promotes synapsis by binding to unpaired sites, and initiates silencing of genes in unpaired zones. Downregulating these genes will result in what has been observed in Drosophila nurse cells, little or no initial synaptonemal complex formation and an apparent withdrawal from subsequent aspects of the meiotic cycle, while maintaining high levels of gene expression. Our observation that largely the same genes are downregulated at the onset of *Xenopus* and mouse nurse cell formation marks this as a nurse cell developmental program that may be ancient and conserved in many animal groups. A list of the genes down-regulated more than threefold in cluster NC1 is given in *SI Appendix*, Table S2 and also includes genes that modulate the cytoskeleton, translation, chromatin, and endocytic trafficking.

The nature and value of such a program can explain a longstanding observation in the study of cysts that has not been well understood. It has been widely observed for >100 y that cysts formed by synchronous divisions produce only one oocyte and 2n-1 nurse cells, a fact known as the “2n rule”(9, 73). The realization that nurse cells abort meiotic chromosome behavior for likely benefit can explain the 2^n^ rule, and explains why early cyst breakage prior to nurse cell commitment can lead to smaller cysts that each generate one oocyte, as seen in mouse oogenesis (43).

### *Xenopus* Cysts Produce Oocytes and Cells with Some but Not All Nurse Cell Features.

The vast majority of insect species, like Drosophila, produce cysts with a majority of nurse cells that transfer materials to the oocyte before turning over. In mammals such as the mouse, about 80% of cyst cells turn over within cysts during prefollicular oogenesis after transferring organelles and cytoplasm to oocytes (43, 44). Here we succeeded in showing by EdU-marking that ~80% of *Xenopus* cyst cells also turn over prior to follicle formation. This dichotomy of cell fates was also clearly revealed by scRNA-seq. The *Xenopus* profile showed a similar picture as in the mouse, in which about 10% of low UMI nurse-like cells from cluster NC1 were found on the UMAP plot inside of the main sequence meiotic cells. These cells are probably transferring some materials through FLS since the growth of UMI in *Xenopus* meiotic cells with stage was very similar to mouse, and shrunken acidified germ cells typical of dying nurse cells in Drosophila and mouse were observed. Clearly, lower vertebrates are not a general exception to the germline cyst paradigm by producing only oocytes (5, 32).

*Xenopus* nurse-like cells almost certainly carry out valuable functions prior to their turnover. Our findings suggest that nurse-like cells may participate in the selective transfer of cytoplasmic materials, including Golgi-derived vesicles, ER, and mRNA, rather than bulk cytoplasm including organelles, possibly via fusome-associated transport mechanisms. They may transfer key protective molecules such as small RNAs and lncRNAs, as suggested by the experiments looking at transfer of *rec8* mRNA. Additionally, our experiments with the photoconvertible protein KikGR, which lacks cellular function, suggest that nonessential proteins are not shared through IBs. Furthermore, we did not detect transfer of organelles in bulk like in mouse or Drosophila cysts: Ring canal diameters did not increase with development, organelles did not accumulate disproportionately in any one cystocyte, and centrioles (marked by centrin-2), remained equally distributed. These results suggest that the oocyte in *Xenopus* may not rely on early bulk organelle transfer within the cyst but instead amplifies organelles during the later LBC stage, when transcription is extremely active (74).

This strategy contrasts with models from Drosophila and mouse, where nurse cells dump large quantities of rejuvenated cytoplasm and organelles into the oocyte during meiotic prophase while still part of a cyst. During meiosis in yeast and Drosophila proteostasis enhancement and organelle competitive selection and repair are thought to help generate and identify highly functional organelles (8, 12, 17, 20, 22, 75). The rejuvenated organelles are then deposited into the oocyte, where they support early embryogenesis. Accordingly, Balbiani bodies in the mouse and Drosophila are transient, dispersing rapidly after organelle transfer. In contrast, *Xenopus* and other lower vertebrates may take a delayed approach: Organelles are not rejuvenated during the cyst stage, and bulk transfer is absent. Instead, the oocyte may gradually improve organelle quality during its long diplotene stage, during which the Bb persists (76). This may explain why the Bb is longer-lasting in *Xenopus*, possibly allowing time for internal organelle selection and maturation.

A related but distinct strategy is observed in some marsupial frogs, where hundreds to thousands of transcriptionally active nuclei coexist within a single oocyte (77, 78). These nuclei contribute biosynthetically, producing RNAs and proteins to support a shared cytoplasm. Eventually, only one nucleus is retained for meiosis, and the others degrade. Although structurally different, this mirrors the *Xenopus* system in which ~80% of cystocytes are eliminated but may transiently serve a nurse-like role. Together, these examples suggest that the key evolutionary function of nurse cells may not always be bulk organelle transfer. Rather, nurse cells—or nurse-like cells—may broadly serve to biosynthetically support the oocyte, either by contributing organelles, transcripts, or cytoplasmic components, depending on the species. The balance between these strategies likely reflects developmental constraints and evolutionary adaptations specific to each lineage.

## Materials and Methods

Detailed descriptions of *X. laevis* husbandry and ovary dissection, scRNA-seq, whole-mount immunofluorescence and FISH, live imaging and photoconversion assays, pharmacological treatments, electron microscopy, and image analysis are provided in *SI Appendix*, *Supplemental Materials and Methods*.

## Supplementary Material

Appendix 01 (PDF)

Movie S1. Crawling-like movements within a germline cyst visualized by Hoechst staining.

Movie S2. **Asynchronous mitotic prophases within a germline cyst**. The arrow indicates two cyst cells that enter mitotic prophase with a delay compared to the others. Hoechst staining.

Movie S3. Fusome-like structure in 8-cell cyst.

Movie S4. Fusome-like structure in 16-cell cyst.

Movie S5. Fusome-like structure in 32-cell cyst.

Movie S6. Fusome-like structure in 32-cell cyst.

Movie S7. Ring canals in different cyst stages.

## Data Availability

The raw and processed scRNAseq data archived at the NIH GEO database under accession number GSE304926 (79).
