# Supplementary material for: Early female germline development in Xenopus laevis: Stem cells, nurse cells, and germline cysts
Source: Proc Natl Acad Sci U S A. 2025 Nov 10;122(46):e2522343122. doi: 10.1073/pnas.2522343122 (PMC12646306; doi:10.1073/pnas.2522343122)
Supplement: Supplementary file 1 — Appendix 01 (PDF) [file pnas.2522343122.sapp.pdf]

PNAS

Supporting Information for
Early female germline development in *Xenopus laevis*: Stem cells, nurse cells and germline cysts

Asya Davidian and Allan C. Spradling

Corresponding author: Allan C. Spradling
Email: spradling@carnegiescience.edu

This PDF file includes:

Supplemental Materials and Methods
Supplemental Tables S1 to S2
Supplemental Figures S1 to S12
Legends for Movies S1 to S7
SI References

Other supporting materials for this manuscript include the following:

Movies S1 to S7

Supplemental Materials and Methods

Experimental model and ethical approval

The experimental model in this study is *Xenopus laevis*. Xenopus tadpoles with 4 limbs (NF59-62), late metamorphic froglets (NF63-65) or sexually mature females were purchased from Xenopus1 company (https://xenopus1.com/). All experiments in this study were performed in accordance with protocols approved by the Institutional Animal Care and Use Committee (IACUC) of the Carnegie Institution of Washington.

Whole-mount staining of Xenopus ovaries

Whole gonads were dissected under a stereomicroscope, briefly washed in L-15 medium, and immediately fixed in freshly prepared 4% paraformaldehyde (PFA) in PBS for 1.5 hr or overnight at room temperature (RT) based on the experimental requirements. Post fixation, tissues were washed in PBS and permeabilized in PBST (PBS with 0.5% Triton X-100 and 0.1% Tween-20) for 1 h then blocked in 10% BSA in PBST for 1 h at RT. Tissues were incubated with primary antibodies for 2 days at RT with mixing, followed by thorough washing in PBST for 1.5 h. Secondary antibodies, DAPI (1 µg/ml), and, if required, Alexa 488-Phalloidin (Thermo Fisher, 1 µg/ml) to visualize cell membranes were applied overnight at RT. After a final 1.5 h wash in PBST, samples were mounted in Vectashield® vibrance antifade mounting medium and imaged using a Leica DIVE confocal microscope.

List of primary antibodies

Antibody	Source	Identifier
Rabbit-anti-ddx4 antibody	Abcam	ab13840
Rabbit-anti-α-Tubulin	Abcam	ab18251
Rabbit-anti-Acetyl-α-Tubulin	Cell Signaling	5335
Mouse-anti-Acetylated-Tubulin	Sigma	T7451
Mouse-Anti-γ-Tubulin (GTU-88)	Sigma	T6557
Mouse-anti-GM130	BD Biosciences	610823
Rabbit-anti-KIF23	Thermo Fisher	PA5-67017
Rabbit-anti-Citrate Synthase	Abcam	ab96600
Rabbit-anti-PDI	Thermo Fisher	PA5-77821
Mouse-anti-Spectrin	Sigma	S3396
Rabbit-anti-alpha-II-Spectrin	Thermo Fisher	PA-35383
Rat-anti-Centrin 2	BioLegend	W16110A
Mouse-anti-Fibrillarin (G-8)	Santa-Cruz	Sc-374022
Mouse-anti-hts (adducin)	DSHB	1B1

LysoTracker staining

Dissected ovaries were cultured in vitro in 1 mL of 50 nM LysoTracker Red (L7528, Thermo Fisher) diluted in L-15 medium supplemented with 5% FBS and 1× Penicillin-Streptomycin, at room temperature for 6 or 15 h. Ovaries were then fixed in 4% PFA/PBS for further analysis.

Live Imaging

For live imaging, to stain nuclei, juvenile ovaries were incubated in L-15 medium with Hoechst 33342 (1:200; ThermoFisher) overnight at RT. The following day, ovaries were rinsed in fresh L-15 medium and mounted in 0.5–1% low-melting agarose in a 35 mm glass-bottom dish (Cat#: D35-20-1.5H) for imaging. After solidification of the agarose, L-15 medium supplemented with 5% FBS and 1× Penicillin-Streptomycin was added to maintain tissue viability during imaging. Z-stack images were acquired using a Leica DIVE confocal microscope with 405 nm excitation, typically using a 40× or 63× oil objective. Ovaries were imaged live within 6 h of staining.

Photoconversion Assay and Live Imaging

To assess cytoplasmic protein exchange between cystocytes, we used juvenile Xenopus laevis ovaries from transgenic frogs expressing the photoconvertible protein KikGR [*Xla.Tg(CAG:KikGR)*] (1). Transgenic froglets (stages NF55–66) were obtained from the National Xenopus Resource (NXR). KikGR fluoresces green under standard excitation and irreversibly converts to red fluorescence upon exposure to UV light. Freshly dissected ovaries were stained overnight at RT with CellMask™ Deep Red plasma membrane dye (#C10046, Thermo Fisher) at 1× concentration in L-15 medium to

visualize cell boundaries. Ovaries were then transferred to a 35 mm glass-bottom dish with a 20 mm microwell and embedded in 0.5–1% low-melting agarose to allow orientation. Once the agarose solidified, L-15 medium supplemented with 5% FBS and 1× Penicillin-Streptomycin was added to cover the tissue. Germline cysts were identified based on CellMask membrane staining. An ROI (region of interest) within a single cystocyte was selected in a cyst and photoconverted using 405 nm UV laser and Z-stack images were acquired for 1 h and 24 h post-conversion using 488 nm, 561 nm, and 647 nm lasers to visualize unconverted and converted KikGR fluorescence and cell membranes, respectively. Z-stacks were captured using a 63× oil immersion objective. At least six photoconversion events were analyzed across multiple ovaries, with consistent results.

Using Fiji, selected z-stacks were processed with Average Intensity Projection to generate a 2D image of each cyst. We then measured the Integrated Density (total intensity) within the region of interest (ROI), adjacent connected cystocytes, and nearby non-cyst cells (follicle). The Integrated Densities of the ROI and neighboring cystocytes were normalized to that of the non-cyst cell/follicle (Figure 5E; white dashed circle marks the ROI, 1–2 indicate neighboring cystocytes, F marks a nearby follicle).

EdU and OPP labeling of ovaries *in vitro*

EdU (5-ethynyl-2'-deoxyuridine; Thermo Fisher) was added to freshly dissected ovaries at 1:500 dilution from stock into L-15 medium supplemented with 5% FBS and 1× Penicillin-Streptomycin. To detect ongoing protein synthesis, O-propargyl-puromycin (OPP; Vector Laboratories) was added at a final concentration of 20 μM. Ovaries were incubated with EdU for at least 1 h at RT or overnight when needed, and with OPP for 2

h at RT. Following incubation, gonads were fixed in 4% PFA in PBS and permeabilized in PBST before proceeding with Click-iT detection as per the manufacturer's protocol.

Lineage tracing with EdU labeling

To determine the developmental timeline and fate of germline cysts, we performed an EdU pulse-chase experiment in Xenopus. A total of 125 metamorphic froglets (NF62–65, including males and females) were injected with EdU (10 μg per froglet) and sacrificed at two-day intervals for gonad dissection up to day 57. Gonads were fixed in 4% PFA in PBS overnight, permeabilized, and processed for EdU detection (ThermoFisher, Cat# C10337) and whole-mount immunofluorescence. The number of EdU-positive germ cell nuclei was manually counted using the Imaris "Spots" function. Because the number of ovarian lobes varied between samples, counts were normalized to the number of lobes in each gonad. The mean number of labeled oocytes per lobe was calculated for each time point (2–4 d, 12 d, 18 d, 33 d, 52–57 d) and then normalized to the mean value at the earliest time point (2–4 days) to determine the relative fraction of EdU-labeled oocytes remaining over time (Fig. 7D).

Nocodazole treatment

Dissected ovaries together with kidney were incubated individually in 24-well plate with Nocodazole (1 μg/mL, SML1665, Sigma) in L-15 supplemented with 5% FBS and 1x Penicillin-Streptomycin for 20 h. Control samples were treated with an equivalent volume of DMSO. Half of each ovary was fixed immediately after drug exposure, considered as 0 h. The remaining halves were washed every 30 minutes for ~6 h in L-15 medium (5% FBS, 1× Pen/Strep) to remove residual drug and then incubated overnight for complete microtubule recovery. The following day, all samples were fixed in 4% PFA

in PBS for 1.5 h at RT. To visualize both microtubules (acetylated tubulin antibody) and ring canals (Kif23 antibody), a 30-minute incubation in detergent solution (1% SDS, 0.5% Tween-20, 50 mM Tris pH 7.4, 1 mM EDTA, 150 mM NaCl) was used following fixation. This treatment enhances ring canal detection, which is otherwise challenging due to their sensitivity to fixation and requirement for antigen retrieval.

Single-molecule whole mount HCR RNA FISH

Customized HCR RNA-FISH probes for *piwil4.S, nefm.S* and *rec8.L* were obtained from Molecular Instruments. Hybridization was performed according to the manufacturer's protocol, with the following modifications. Ovaries were fixed in 4% PFA in PBS for 1.5 h at RT, dehydrated through a series of methanol treatment and stored at –20 °C until used. Rehydrated ovaries were permeabilized for 30 minutes in detergent solution (1% SDS, 0.5% Tween-20, 50 mM Tris pH 7.4, 1 mM EDTA, 150 mM NaCl). Tissues were pre-hybridized and then incubated with probes at a final concentration of 10 nM overnight at 37 °C. After washes and the amplification step, samples were washed in 5× SSCT (5× SSC with 0.1% Tween-20) containing DAPI (1 µg/ml), mounted in Vectashield antifade, and imaged.

Combined HCR RNA FISH and immunofluorescence

To combine HCR RNA-FISH with immunofluorescence, we implemented the following modifications to the standard protocol. Ovaries were fixed in 4% PFA for 1.5 h at RT, washed thoroughly in PBST, and incubated in detergent solution for 30 minutes to permeabilize the tissue. Immunostaining was performed using primary antibodies against Kif23 and acetylated tubulin. After secondary antibody incubation and washes, the tissue was fixed in 2% PFA in PBS for 10 min, followed by several PBST washes. Gonads were

then either dehydrated through a methanol series or directly processed for HCR RNA-FISH, following the protocol described above.

Whole-mount telomere DNA FISH

A Cy3-labeled telomere-specific DNA probe (/5Cy3/TTAGGG repeated 7×) was used to label telomeres. After 1.5 h fixation, samples were permeabilized in PBST for 1 h, followed by proteinase K treatment (10 µg/ml, 10 min). Tissues were washed, fixed in 2% PFA in PBS for 10 min and rinsed in PBST. Pre-hybridization was performed in buffer containing 50% formamide, 5× SSC, 0.1% Tween-20, and 0.5 mg/ml tRNA for 1 h or overnight. Probes were diluted to 2–5 ng/µl in hybridization buffer and co-denatured with the sample at 75 °C for 5 min, followed by overnight incubation at 37 °C. Post-hybridization washes were performed stepwise using decreasing concentrations of pre-HB in 2× SSC, then 0.2× SSC and 2× SSC (with 0.1% Tween-20). Samples were stained with DAPI, mounted in Vectashield antifade, and imaged under a confocal microscope.

Single-Cell ovary dissociation and sequencing

Ovaries from juvenile Xenopus were dissected at developmental stages between NF62–66. Dissections were performed in L-15 medium at RT. A fresh collagenase solution was prepared in L-15 containing 3 mg/ml Collagenase I (Gibco 17018029), 2 mg/ml Collagenase IV (Gibco 17104019), 3 mg/ml Collagenase II (Sigma C6885), and 1 mM $CaCl_2$ Ovaries were chopped into small pieces in collagenase solution and transferred into low-adhesion 1 ml tubes. Tissues were digested for ~40 min at RT with rotation. Cells were pelleted at 300 × g for 3 min, washed in PBS, and digested with 1 ml TrypLE Express (Gibco) for 10 min at RT. The reaction was quenched by adding 10% FBS. Cells were washed twice with cold PBS containing 1% BSA, resuspended in 1 ml

PBS with 1% BSA, and filtered through 70 μm and then 40 μm cell strainers to remove clumps. Cells were pelleted at 400 × g for 5 min, resuspended in ~50 μl PBS, and viability was assessed using automated cell counter (Countess™ 3 FL, ThermoFisher). Cell viability was >95% with minimal clumping. Approximately 10,000 live cells per sample were loaded onto the 10X Genomics Chromium system following the manufacturer's protocol. Libraries were sequenced on an Illumina NextSeq 500 and processed using Cell Ranger v8.0.1 with default settings.

Single-cell RNA-seq analysis

Single-cell RNA-seq data from three independent *Xenopus laevis* juvenile ovaries were processed using Seurat v5.1.0. Each sample was converted into a Seurat object and merged using CCA-based integration. Low-quality cells were excluded based on gene count (<100 or >8000 genes) and mitochondrial gene content (>1.5%). Gene expression was normalized (LogNormalize method), and variable features were identified (2000 genes, VST method). Data were scaled, principal component analysis (PCA) was performed, and the top 20 PCs were used for clustering (resolution = 2) and UMAP visualization. After integration using IntegrateLayers() with CCA, the dataset was reclustered using the first 20 dimensions of the integrated reduction (resolution = 1) and visualized with UMAP. Germline clusters were selected based on expression of germ cell markers and UMAP position, then subsetted for reanalysis. The germline subset was normalized, scaled, and reanalyzed using PCA, clustering (resolution = 0.5), and UMAP using 30 dimensions of the integrated reduction.

Electron microscopy

Ovarian tissue was fixed in 2% PFA/2.5% glutaraldehyde and in 0.1 M cacodylate buffer (pH 7.4) at RT for 1–2 h, then stored at 4°C. Samples were post-fixed in 1% osmium tetroxide, dehydrated through a graded ethanol series, and embedded in epoxy resin. Ultrathin sections (70–90 nm) were cut using an ultramicrotome, collected on copper grids, stained with uranyl acetate and lead citrate, and imaged with a transmission electron microscope (Hitachi TEM HT7800).

Partial Ovariectomy

Partial ovariectomy was performed as described in *Xenopus* oocyte isolation protocols (2).

Image analysis and 3D reconstruction using Imaris

Imaris software (Bitplane) was used for quantitative and three-dimensional analyses of confocal image stacks. To determine the exact number of cells within germline cysts, the "Cell" module was used to segment nuclei and measure EdU-labeled cells in 3D. The "Spots" function was applied to count EdU-positive nuclei across different time points following EdU injection. For structural visualization of the fusome, the "Surface" module was used to reconstruct acetylated tubulin-labeled fusomes in three dimensions, allowing assessment of their architecture and connectivity within germline cysts.

Supplemental Tables

Table S1. Marker genes for scRNAseq cluster identification.

# of cluster	Name of cluster	Marker genes	Function of marker genes	References	Morphology
1	**GSC**	*piwil4.S*<br>*chgb.L*<br>*dnmt3a*	*piwil4* - transposon silencing;<br>*chgb* - secretory granules of neuroendocrine cells;<br>*dnmt3a* - DNA methylation	(3, 4)	Large cells with polymorphic nucleus, dispersed chromatin, and prominent mitochondrial cloud.
2	**G2/M**	*ccnb3.S*<br>*cdk1.L*<br>*melk.L*<br>*mki67.S*	Proliferation markers	(5, 6)	Dividing cysts
3	**Oogonia (Oog)**	*cyp26a1.S*	Meiotic entry repressor	(7)	Cysts with 2-, 4-, 8-, 16-, 32-cells before meiosis.
4	**Pre-Meiotic S (P-M S)**	*rec8.L*	Meiotic recombination protein	(8)	Often EdU-positive, undergoing pre-meiotic S phase; morphologically similar to oogonia cysts.
5	**Leptotene-Zygotene (L-Z)**	*dmc1.L*<br>*spo11.L*<br>*sycp3.L*<br>*hormad1.S*	Genes involved in meiotic double-strand break formation, recombination, and synaptonemal complex assembly	(9)	Cysts with telomere bouquets, nucleolus repositioning (1–2), and initiation of rDNA amplification.
6	**Early Pachytene (EP)**	*syce2.L*<br>*hormad1.S*	Synaptonemal Complex Central Element Protein 2 - an essential structural component of the central element of the synaptonemal complex.	-	Prominent rDNA cap opposite the telomeres.
7	**Late Pachytene (LP)**	*syce2.L*<br>*figla.L*	*syce2* - the central element of the synaptonemal complex;<br>*figla* - oocyte-specific transcription factor	(10)	Increased cell/nuclear size; fully developed rDNA cap.
8	**Early Diplotene (ED)**	*figla.L*<br>*velo1.L*<br>*vegt.L*	*figla* - oocyte-specific transcription factor;<br>*velo1* - marker of Balbiani body;	(10–13)	Oocytes separated from cyst, forming primordial follicles; rDNA amplification

			vegt - transcription factor required for both mesoderm and endoderm formation in the embryo		ceased, telomeres dispersing.
9	**Stage 1 Follicle (St1)**	*zp4.L* *velo1.L* *vegt.L*	*zp4* - zona pellucida - the extracellular matrix surrounding oocytes; *velo1* - marker of Balbiani body; *vegt* - transcription factor required for both mesoderm and endoderm formation in the embryo	(13)	Enlarged follicles with large germinal vesicles and telomeres detached from the nuclear membrane; forming lampbrush chromosomes.
10	**NC3**	-	-	-	-
11	**NC1**	-	-	-	-
12	**NC2**	-	-	-	-

Information regarding marker genes in each scRNAseq cluster (cluster number) is given. Names are based on the meiotic or other developmental stage deduced from its gene expression. The name and function of indicated marker genes that validate stage assignments are given, along with reference(s). The last column includes a brief description of the stage to assist in identification.

Table S2. Genes downregulated in early nurse cells (NC1).

Xenopus	Mouse	LZ avg exp	NC1 avg exp	NC1 / LZ
faim2	Faim2	7.02	0.78	0.11
sycp3	Sycp3	4.65	0.57	0.12
dmc1	Dmc1	5.18	0.66	0.13
hormad1	Hormad1	10.11	1.50	0.15
sycp1	Sycp1	9.68	1.61	0.17
macroh2a2	Macroh2a2	3.05	0.65	0.21
syce2	Syce2	6.71	1.47	0.22
arl3	Arl3	2.51	0.55	0.22
spata22	Spata22	8.50	1.93	0.23
ano1	Ano1	2.62	0.61	0.23
fkbp10	Fkbp10	3.73	0.88	0.24
agtrap	Agtrap	5.75	1.44	0.25
dazl	Dazl	19.69	5.01	0.25
ccdc63	Ccdc63	1.86	0.51	0.27
syce3	Syce3	6.10	1.71	0.28
ccdc63	Ccdc63	1.88	0.54	0.29
hmces	Hmces	5.32	1.50	0.29

A list of genes (Xenopus) and their mouse orthologs (Mouse) regulated in early nurse cells (NC1) relative to the leptotene-zygotene (L-Z, cluster 5) from which they derive as they leave the main circle of germ cell development. Average gene expression is given as well as the ratio of NC1/L-Z. Genes are listed from smallest to largest NC1/L-Z ratio (all genes from among 12,154 are shown in which NC1/L-Z <0.3 and L-Z >1 and NC1>0.5.

Supplemental Figures

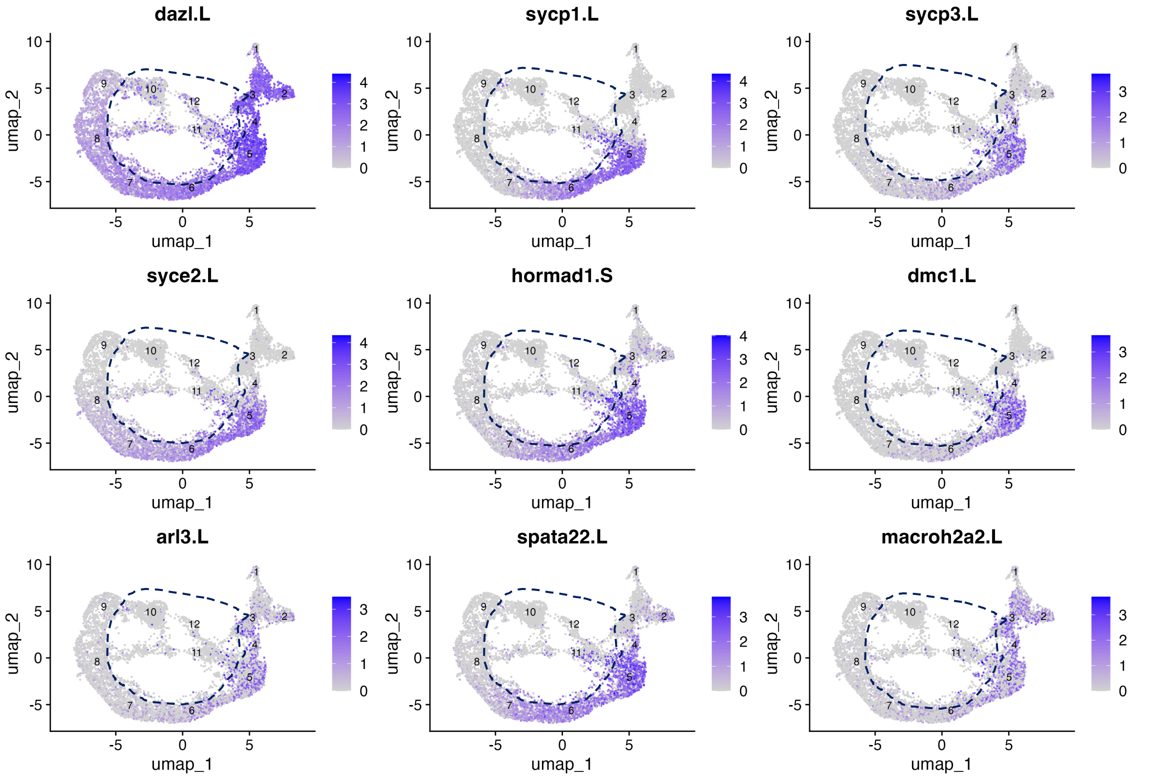

Fig. S1. Genes preferentially downregulated in nurse-like cells compare to meiotic cells in outer ring. Feature plots showing the expression of some representative meiotic genes that are downregulated in nurse-like cell clusters compared to germ cells of the same developmental stage. Each dot represents a single cell, with darker colors indicating higher expression levels. Cells along the main developmental trajectory (outlined with a red dashed line) generally exhibit stronger expression of these genes than cells in the nurse cell clusters (outlined in blue). Refer to Figures 1A and 7E for spatial orientation of these cell groups within the UMAP. **Cluster identity**: 1 - "GSC", 2 - "G2/M cyst", 3 - "Oogonia cyst", 4 - "Pre-Meiotic S cyst", 5 - "Leptotene-Zygotene cyst", 6 - "Early Pachytene cyst", 7 -"Late Pachytene cyst", 8 - "Early Diplotene", 9 - "Stage 1 Follicle", 10 - "NC3"; 11 - "NC1", 12 - "NC2".

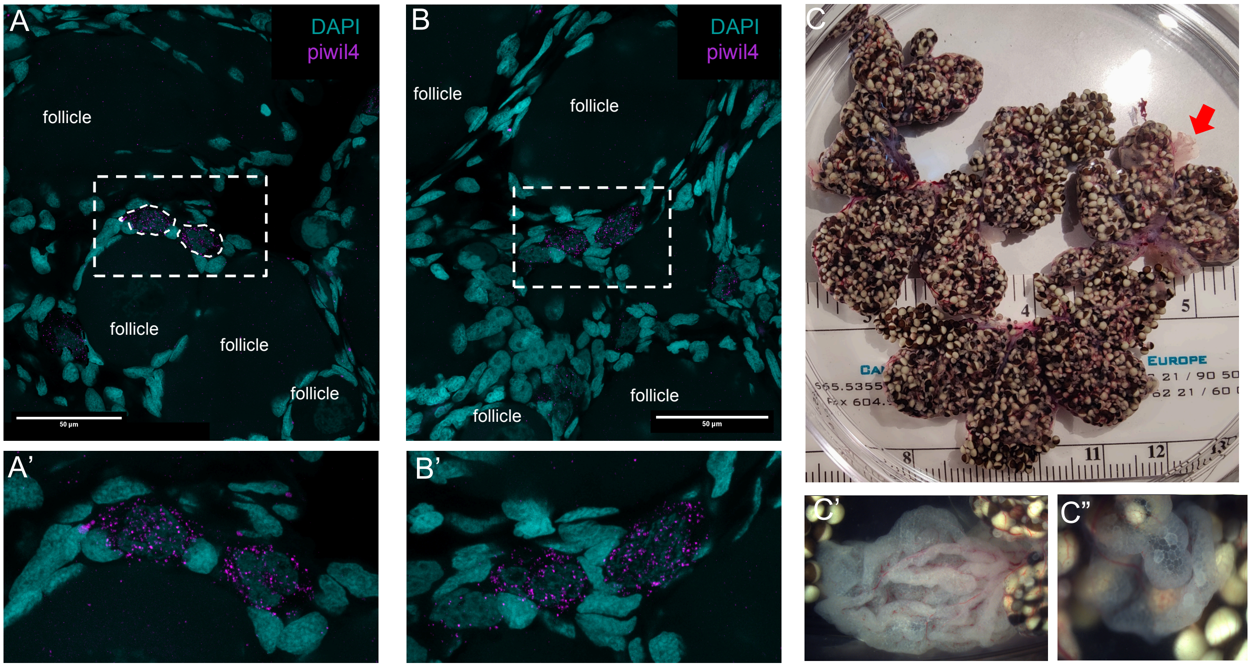

Fig. S2. Germline stem cells in the adult Xenopus ovary. **A–B** – Germline stem cells (GSCs) located on the epithelial surface of the adult *Xenopus* ovary, marked by *piwil4* RNA expression (magenta). The dashed boxes in A and B are shown as magnified images in A’ and B’. **C** – Adult ovary pair approximately five years after partial ovariectomy. Red arrow indicates a regenerating ovarian lobe. **C′–C″** – Close-up of the regenerating lobe from panel C (red arrow), showing developing white-translucent follicles.

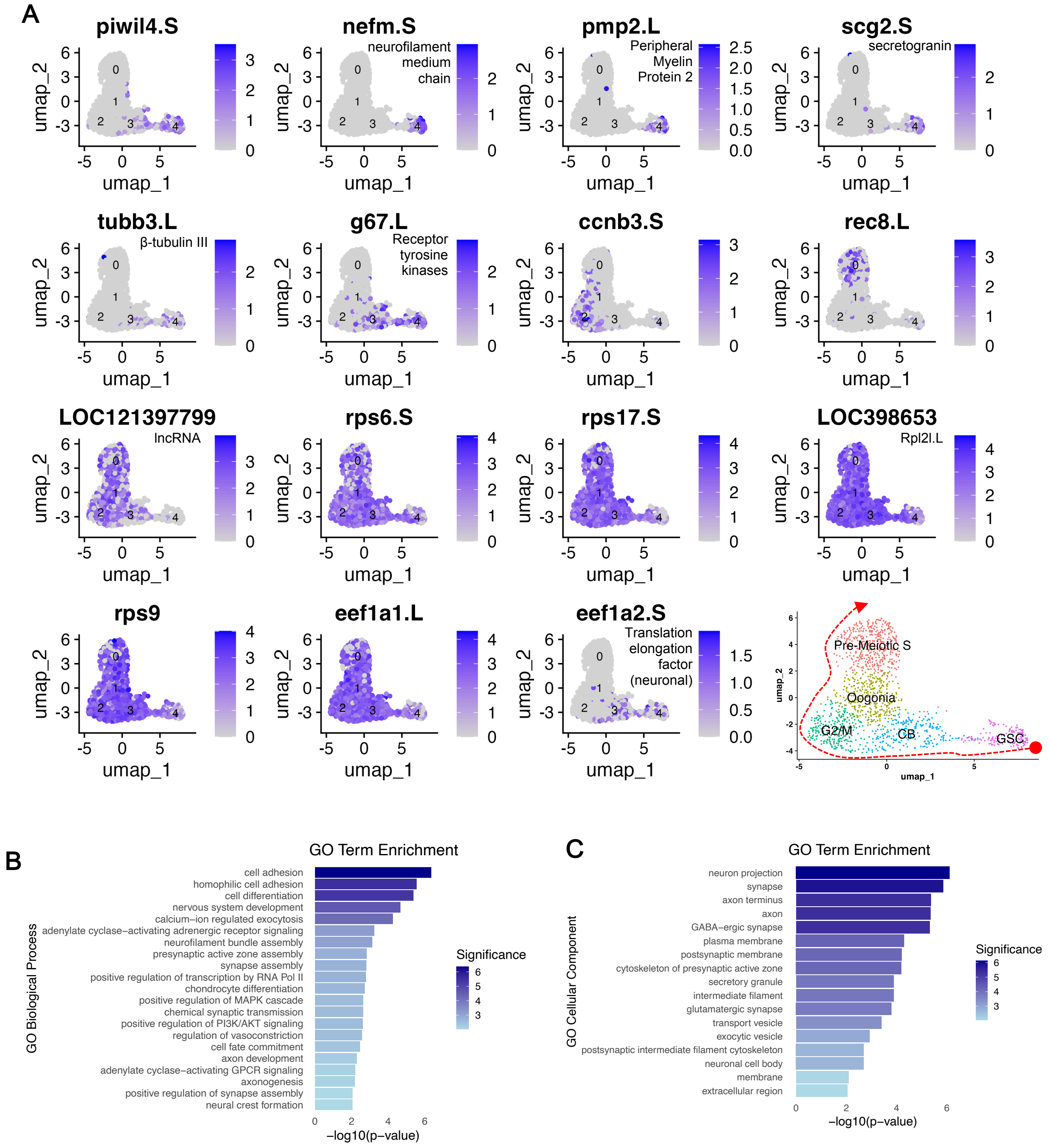

Fig. S3. Expression features of early germline clusters from GSCs to pre-meiotic S-phase. A – Early-stage germline clusters from Figure 1A (#1 – GSC, #2 – G2/M cyst, #3 – oogonia) were subsetted and re-clustered in Seurat. The quiescent GSC cluster (#4) expressed neuronal genes (*nefm.S*, *pmp2.L*, *scg2.S*, *tubb3.L*) alongside *piwil4.S*. The

putative cystoblast (CB) cluster (#3) showed upregulation of ribosomal genes (*rps6.S*, *rps17.S*, *rps9*, *LOC398653* [*rpl2.L*, 60S ribosomal protein], *eef1a1.L*) and the lncRNA *LOC121397799*, which were expressed at lower levels in GSCs (#4). Notably, *eef1a2.S*, a neuronal-specific translation elongation factor, was restricted to clusters #4 and #3, suggesting a specialized translational program in early germ cell. Cluster #2 likely represents dividing oogonia, marked by *ccnb3* expression. Cluster #0 corresponds to pre-meiotic S-phase cells, characterized by *rec8.L* expression. Cluster #1 lacked significantly enriched unique markers and is likely composed of non-dividing interphase oogonia. **B–C** – GO term enrichment (NCBI DAVID) for Biological Process and Cellular Component categories based on the top 500 upregulated genes in the GSC cluster.

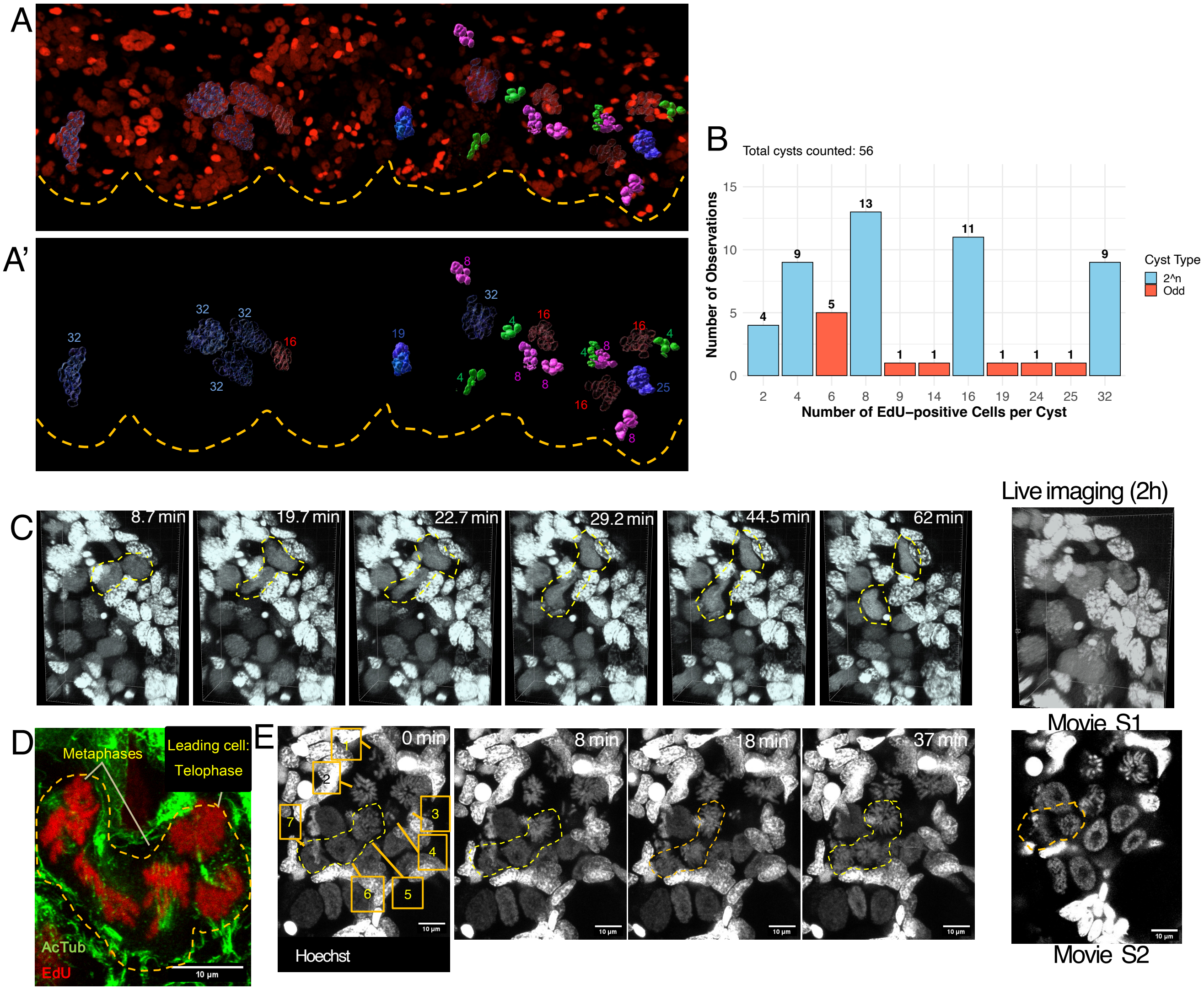

Fig. S4. Cyst dynamics and asynchrony. A–A' – Region of a juvenile *Xenopus* ovary with 6 lobes (dashed outline) after ~6 hours of EdU incubation (red). The full confocal z-stack is displayed in Imaris, showing the spatial arrangement of germline cysts. Some cysts were 3D reconstructed using the Imaris "Cell" module. **A'** – EdU-positive cysts were analyzed using Imaris "Cell" module to quantify the number of cells per cyst. **B** – A total of 56 cysts from multiple juvenile ovaries, including those shown in (A), were analyzed. **C** – Live imaging over 2 h (Movie S1) showing sister oogonia crawling within the ovary. Hoechst staining reveals germline nuclei as less intensely stained compared to somatic nuclei. **D** – Asynchronously dividing 4-cell cyst labeled with EdU (red) and

acetylated tubulin (green); the “leading” cell is in telophase with a visible midbody, while the remaining cystocytes are in metaphase. **E** – Live imaging over 2 h (Movie S2) shows asynchronous mitotic prophases within a germline cyst (Hoechst). Cells #1–4 are in mitotic prophase at 0 min; cell #5 enters prophase at 8 min; cells #6 and #7 enter at 18 min.

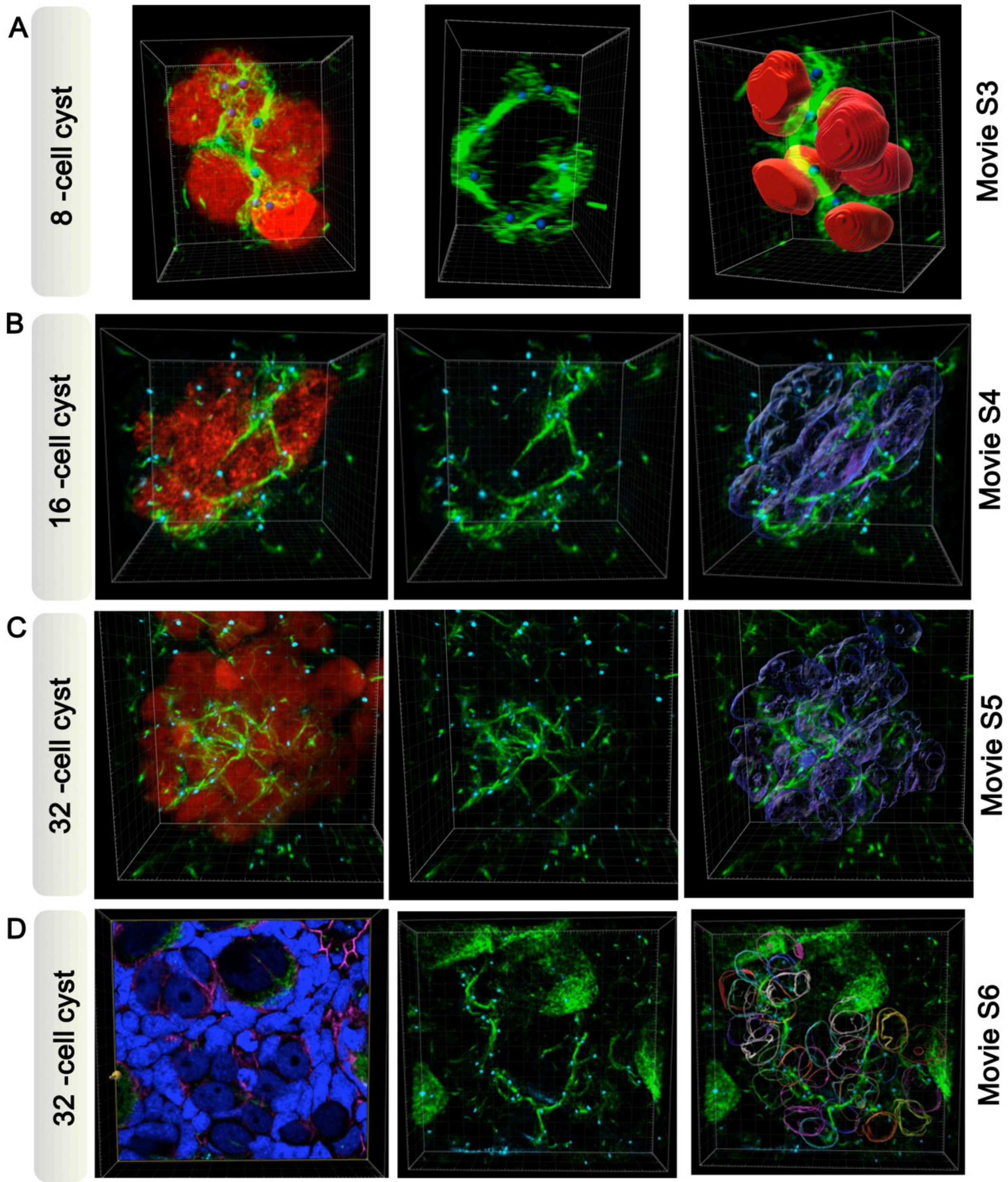

Fig. S5. FLS in 8-, 16-, and 32-cell oogonia cysts visualized in Imaris as 3D (movies). **A–C** – 3D movies (S3-S6) of cysts with 8 (A), 16 (B), and 32 (C) cells labeled with EdU (red), acetylated microtubules (green), centrioles (cyan), and nuclear surfaces (red or blue). **D** – 32-cell cyst showing FLS visualized by acetylated tubulin (green), centrioles (cyan), cell membranes (magenta), DAPI (blue), and segmented nuclear surfaces (varied colors).

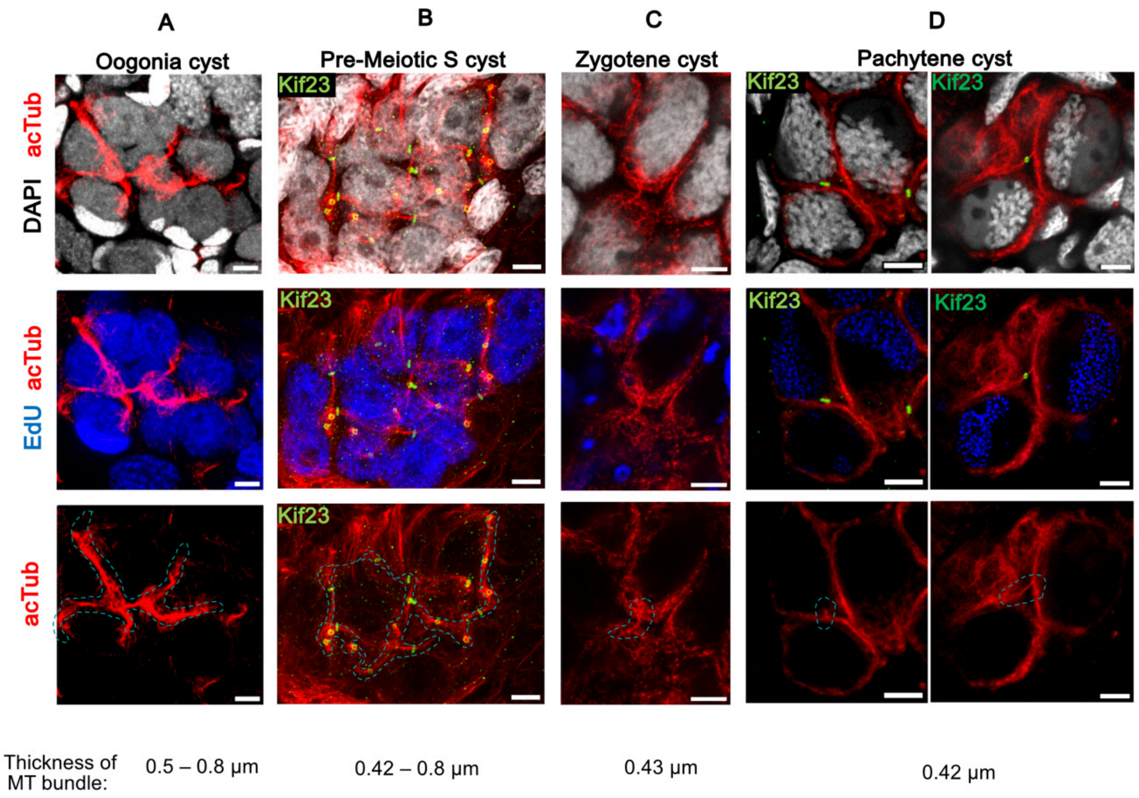

Fig. S6. FLS during germline cyst development from oogonia to pachytene stages. The appearance of FLS changes across stages – from oogonia (**A**), pre-meiotic S (**B**), and zygotene (**C**) to pachytene (**D**) – but microtubule bundles spanning intercellular bridges remain similar in size, as ring canal diameter does not change significantly. Whole-mount ovaries were stained with EdU (blue), acetylated microtubules (red), Kif23 (green), and DAPI (gray). Scale bar 5 µm.

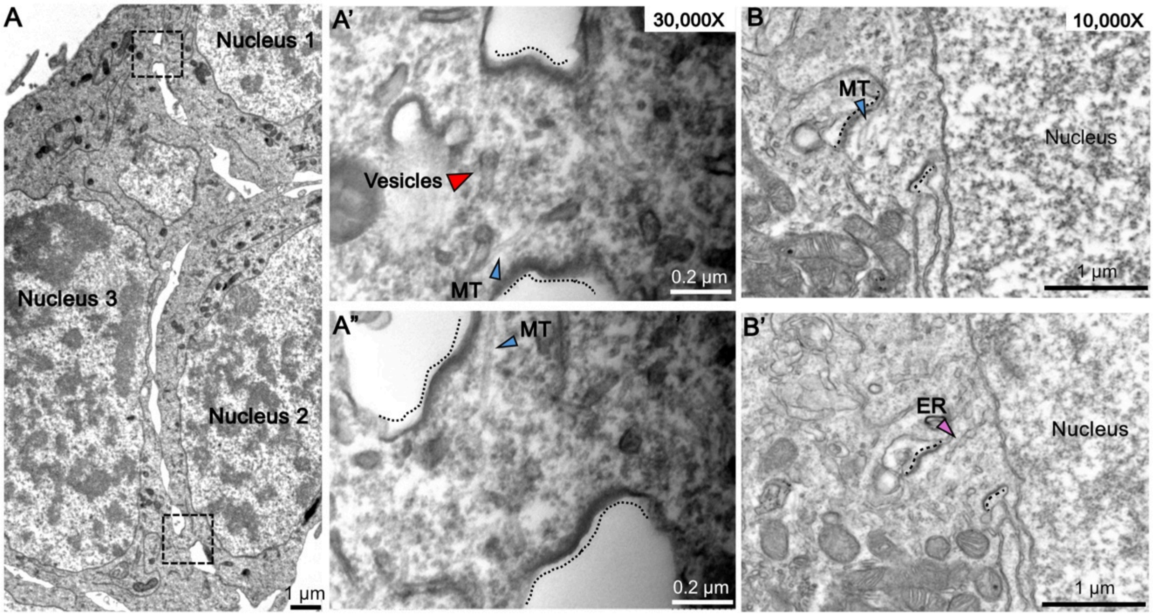

Figure S7. Ring canals with microtubules, vesicles, and ER-like structures under electron microscopy. A – Three meiotic sister cells connected via two ring canals (dashed boxes). **A'–A''** – Zoomed-in views of ring canals from panel A, showing microtubules (MT, blue arrowhead) and vesicles (red arrowhead) within the ring lumen (black dashed lines). **B–B'** – Serial sections of the same ring canal where on **B** – ring lumen filled with MTs and on **B''** – ER-like structure (pink arrowhead) passing through the ring canal (dashed line).

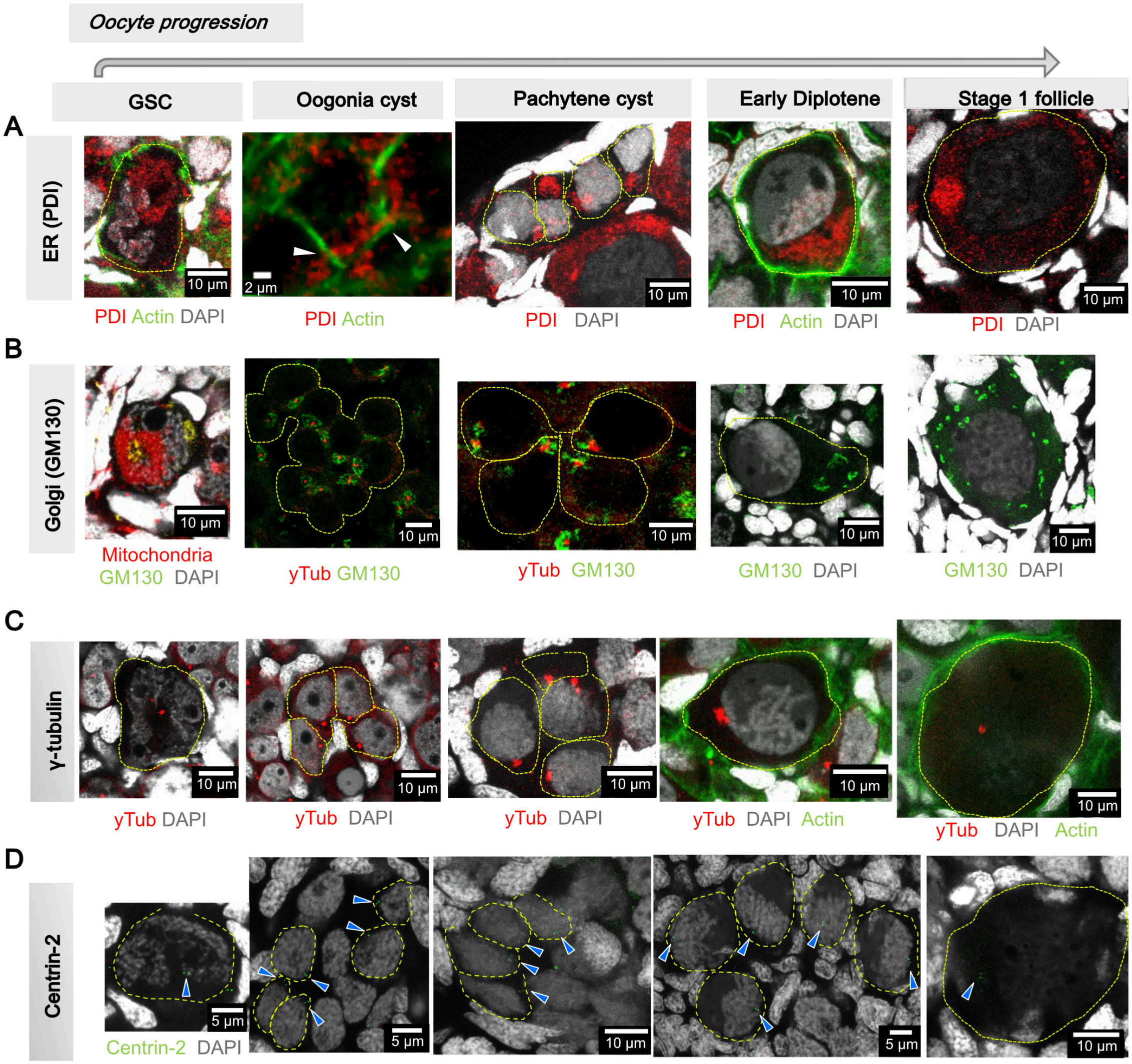

Figure S8. Organelle dynamics during germline cyst development. **A** – An enriched endoplasmic reticulum (ER) cluster, stained with PDI (red), is present in GSCs. In oogonial cysts ER often colocalizes with acetylated microtubules. In meiotic (pachytene) cysts and early diplotene, ER accumulates near the centrosome at the vegetal pole. In stage I follicles, ER localizes to the Balbiani body. DAPI (gray), ER (PDI, red), actin (Phalloidin, green). **B** – Golgi apparatus, stained with GM130, appears as a large ring in GSCs, and as centrosome-centered rings in each cystocyte of oogonia cysts, forming a rosette-like pattern. In pachytene cysts, Golgi structures increase in size. In early diplotene, they may appear fragmented, and by stage I, Golgi is dispersed throughout the

cytoplasm as small stacks. DAPI (gray), Golgi (GM130, green), γ-tubulin (red). **C** – γ-tubulin signal increases from GSCs through meiotic cyst stages, peaking in pachytene, and then declines in stage I follicles. DAPI (gray), Actin (green), γ-tubulin (red). **D** – Centrin-2 staining reveals two centriolar dots per germline cell across all stages (blue arrowheads), from GSCs to stage I follicles. DAPI (gray), Centrin-2 (green).

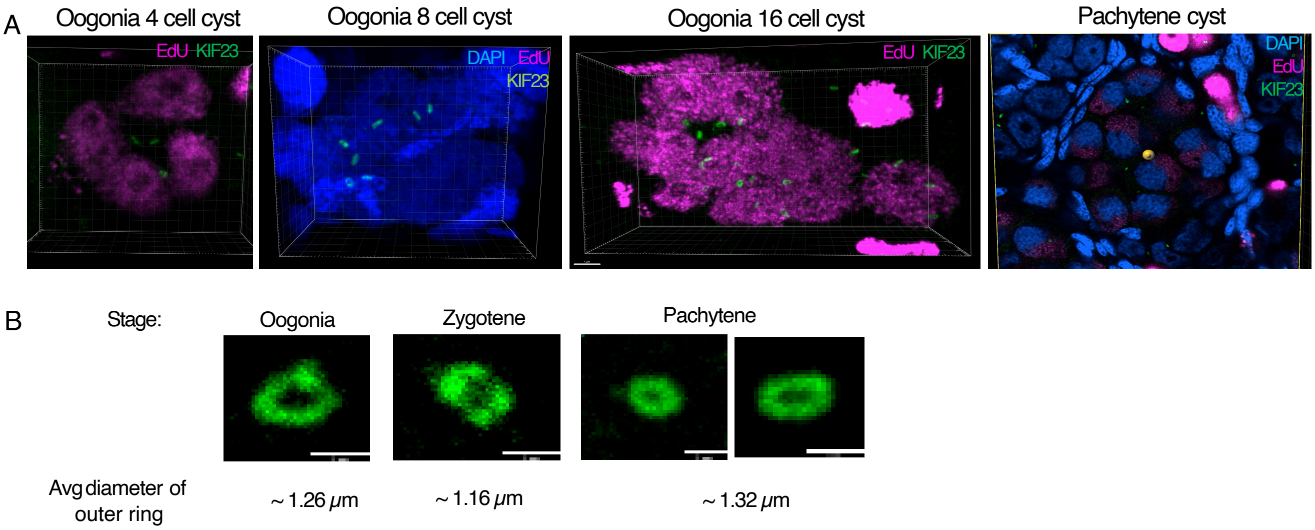

Figure S9. Ring canals during different cyst stages (Movie S7). A - Movies (S7) of z-stack of cysts labeled with EdU (magenta), Kif23 (green), DAPI (blue). **B** – Average diameter of single ring canals stained in green (Kif23) across different stages within a cyst. Scale bar 1 μm.

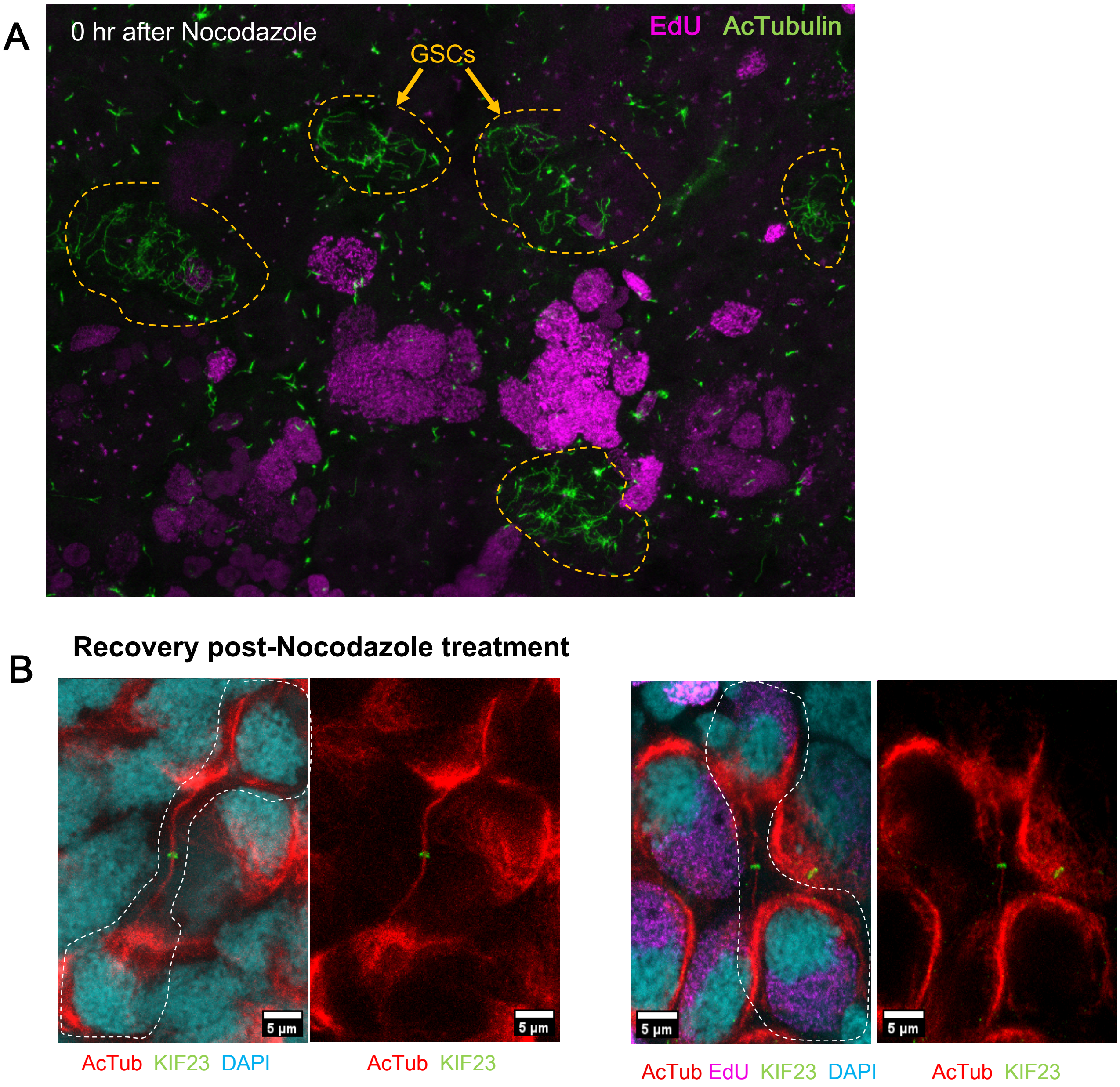

Figure S10. Effect of Nocodazole treatment on cyst microtubules. A – 0 hour after nocodazole: EdU (magenta), acetylated MT (green). GSCs with resistant MT are encircled with dashed lines (maximum projection of several optical sections). **B** – Showing recovered and highly elongated MT between two cystocytes within a cyst (encircled in white dashed lines).

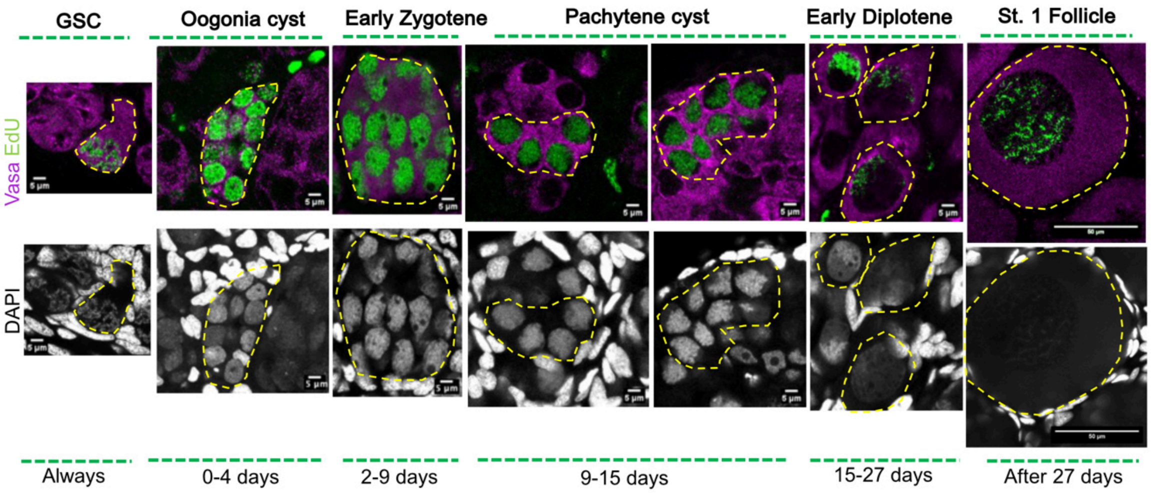

Figure S11. Timeline of germline cyst development by EdU pulse. Froglets were injected with EdU to understand the developmental timeline of germline cysts. As shown in figure, some of the GSCs are positive for EdU at any time point whereas some remains negative indicating active and quiescent stages of GSCs. Oogonial cysts appeared 0–4 days after the EdU pulse, leptotene–zygotene cysts at 2–9 days, pachytene cysts at 9–15 days, early diplotene cysts at 15–27 days, and stage I follicles after 27 days. EdU (green), Vasa (magenta), DAPI (gray).

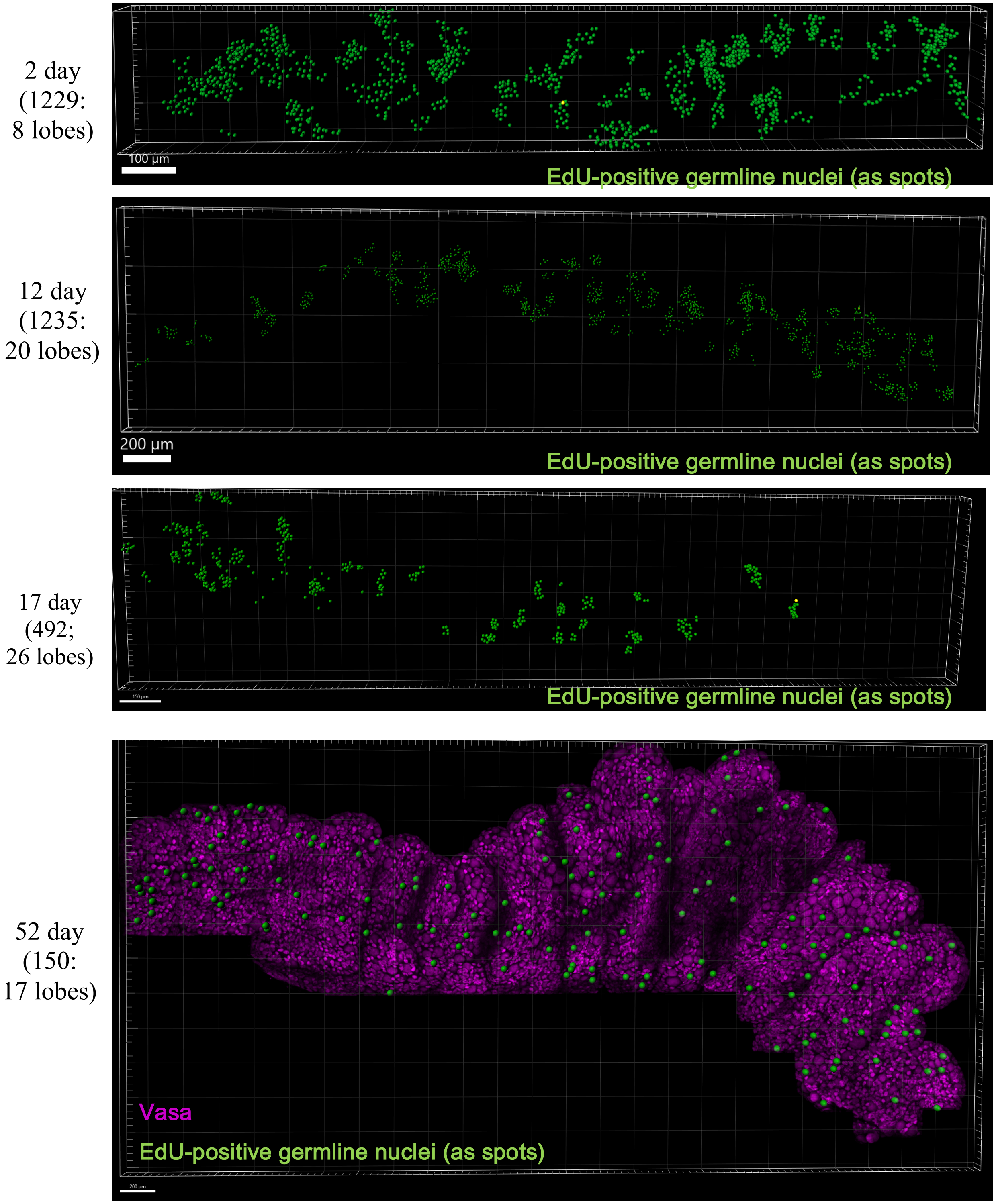

Figure S12. Examples of quantification of EdU-positive germline nuclei from EdU-pulsed ovaries. Using Imaris, the 'Spot' function was applied to manually count EdU-positive germline nuclei (green) in ovaries at various time points after the EdU pulse. Vasa (magenta).

Legends for Movies

Movie S1. Crawling-like movements within a germline cyst visualized by Hoechst staining.

Movie S2. Asynchronous mitotic prophases within a germline cyst. The arrow indicates two cyst cells that enter mitotic prophase with a delay compared to the others. Hoechst staining.

Movie S3. Fusome-like structure in 8-cell cyst.

Movie S4. Fusome-like structure in 16-cell cyst.

Movie S5. Fusome-like structure in 32-cell cyst.

Movie S6. Fusome-like structure in 32-cell cyst.

Movie S7. Ring canals in different cyst stages.

SI References

1. P. Tandon, Y. V. Miteva, L. M. Kuchenbrod, I. M. Cristea, F. L. Conlon, Tcf21 regulates the specification and maturation of proepicardial cells. *Development* **140**, 2409–2421 (2013).

2. H. L. Sive, R. M. Grainger, R. M. Harland, Isolation of Xenopus Oocytes. *Cold Spring Harb. Protoc.* **2010**, pdb.prot5534 (2010).

3. J. Guo, *et al.*, The adult human testis transcriptional cell atlas. *Cell Res.* **28**, 1141–1157 (2018).

4. Y. Liu, *et al.*, Single-cell transcriptome reveals insights into the development and function of the zebrafish ovary. *eLife* **11**, e76014 (2022).

5. C. Badouel, I. Chartrain, J. Blot, J.-P. Tassan, Maternal embryonic leucine zipper kinase is stabilized in mitosis by phosphorylation and is partially degraded upon mitotic exit. *Exp. Cell Res.* **316**, 2166–2173 (2010).

6. P. Lara-Gonzalez, *et al.*, Cyclin B3 is a dominant fast-acting cyclin that drives rapid early embryonic mitoses. *J. Cell Biol.* **223**, e202308034 (2024).

7. R. Feng, *et al.*, Retinoic acid homeostasis through aldh1a2 and cyp26a1 mediates meiotic entry in Nile tilapia (Oreochromis niloticus). *Sci. Rep.* **5**, 10131 (2015).

8. Y. Watanabe, S. Yokobayashi, M. Yamamoto, P. Nurse, Pre-meiotic S phase is linked to reductional chromosome segregation and recombination. *Nature* **409**, 359–363 (2001).

9. F. Carofiglio, *et al.*, Repair of exogenous DNA double-strand breaks promotes chromosome synapsis in SPO11-mutant mouse meiocytes, and is altered in the absence of HORMAD1. *DNA Repair* **63**, 25–38 (2018).

10. L. Liang, S. M. Soyal, J. Dean, FIGα, a germ cell specific transcription factor involved in the coordinate expression of the zona pellucida genes. *Development* **124**, 4939–4947 (1997).

11. M. Claußen, T. Pieler, Xvelo1 uses a novel 75-nucleotide signal sequence that drives vegetal localization along the late pathway in *Xenopus* oocytes. *Dev. Biol.* **266**, 270–284 (2004).

12. Divyanshi, J. Yang, Germ plasm dynamics during oogenesis and early embryonic development in Xenopus and zebrafish. https://doi.org/10.1002/mrd.23718.

13. F. Stennard, G. Carnac, J. B. Gurdon, The Xenopus T-box gene, Antipodean, encodes a vegetally localised maternal mRNA and can trigger mesoderm formation. *Development* **122**, 4179–4188 (1996).
